# Diagnostic classification of mild cognitive impairment in Parkinson's disease using subject-level stratified machine-learning analysis

**DOI:** 10.3389/fnagi.2025.1687925

**Published:** 2025-10-22

**Authors:** Jing Wang, Yanfang Chen, Xiao Xie, Pengwei Wang, Hang Hu, Hongfang Han, Lihan Wang, Li Zhang

**Affiliations:** ^1^School of Computer and Information Technology, Xinyang Normal University, Xinyang, China; ^2^Henan Key Laboratory of Education Big Data Analysis and Applications, Xinyang Normal University, Xinyang, China; ^3^School of Computer Science and Information Engineering, Shanghai Institute of Technology, Shanghai, China; ^4^Shandong Provincial Key Laboratory of Integrated Traditional Chinese and Western Medicine for Prevention and Therapy of Ocular Diseases, Jinan, China; ^5^Shandong Academy of Eye Disease Prevention and Therapy, Jinan, China; ^6^School of Early-Childhood Education, Nanjing Xiaozhuang University, Nanjing, China

**Keywords:** mild cognitive impairment, Parkinson's disease, machine learning, stratified sampling, Bayesian optimization, feature importance

## Abstract

**Background:**

The timely identification of mild cognitive impairment (MCI) in Parkinson's disease (PD) is essential for early intervention and clinical management, yet it remains a challenge in practice.

**Methods:**

We conducted an analysis of 3,154 clinical visits from 896 participants in the Parkinson's Progression Markers Initiative (PPMI) cohort. Participants were divided into two groups: cognitively normal (PD-NC, MoCA ≥ 26) and MCI (PD-MCI, 21 ≤ MoCA ≤ 25). To ensure no visit-level information leakage, subject-level stratified sampling was employed to split the data into training (70%) and hold-out test (30%) sets. From an initial set of 12 routinely assessed clinical features, seven were selected using least absolute shrinkage and selection operator (LASSO) logistic regression: age, sex, years of education, disease duration, UPDRS-I, UPDRS-III, and Geriatric Depression Scale (GDS). Four machine learning models—logistic regression (LR), support vector machine (SVM), random forest (RF), and extreme gradient boosting (XGBoost)—were trained using subject-level stratified 10-fold cross-validation with Bayesian optimization. Probabilistic outputs were dichotomized using three thresholding strategies: default 0.5, F1-score maximization, and Youden index maximization.

**Results:**

On the independent test set, SVM achieved the highest overall performance with AUC-ROC of 0.7252 and AUC-PR of 0.5008. LR also performed competitively despite its simplicity. RF achieved the top performance in sensitivity, reaching 0.8150. Feature importance analysis consistently highlighted age, years of education, and disease duration as the most informative predictors for distinguishing PD-MCI. Additionally, more stringent site-level split validation yielded slightly decreased overall performance, with LR showing improved AUC-PR. Importantly, the core feature importance ranking remained largely consistent across validation strategies.

**Conclusion:**

This study developed and validated robust machine learning models for PD-MCI classification using standard clinical assessments alone. Through subject-level or site-level stratified cross-validation combined with Bayesian optimization, we achieved rigorous model evaluation while minimizing overfitting risk. These findings demonstrate the potential for implementing data-driven, interpretable diagnostic tools to enhance early cognitive impairment screening in routine PD care.

## 1 Introduction

Parkinson's disease (PD) is a common degenerative disease of the central nervous system, pathologically characterized by progressive loss of dopaminergic neurons in the substantia nigra and formation of Lewy bodies (Poewe et al., 2017). The global prevalence of PD has been continuously increasing since the 1980s, with an accelerating trend observed in the recent two decades (Zhu J. et al., 2024). As of 2021, approximately 11.8 million people worldwide were living with PD (Luo et al., 2025; Steinmetz et al., 2024). By 2050, the global burden is projected to reach 25.2 million PD patients (predicted range: 21.7 to 30.1 million) (Su et al., 2025). The clinical manifestations of PD are not limited to motor symptoms such as resting tremor, bradykinesia, muscle rigidity, and postural gait disorders, but also include a series of non-motor symptoms, including sleep disorders, olfactory dysfunction, autonomic dysfunction, psychiatric symptoms, and cognitive impairment (Chaudhuri et al., 2006; Postuma et al., 2015).

Among the numerous non-motor symptoms, cognitive impairment has a particularly substantial impact on the quality of life and disease prognosis of PD patients, representing one of the main causes of disability and dependence (Goldman et al., 2018b; Aarsland et al., 2021). PD presents a broad spectrum of cognitive impairment, ranging from subjective cognitive decline (SCD) and mild cognitive impairment (PD-MCI) to Parkinson's disease dementia (PDD) (Goldman et al., 2018a).

PD-MCI is recognized as a significant risk factor and a potential prodromal stage for PDD (Litvan et al., 2012), with a prevalence of approximately 20%–26% in PD patients without dementia (Aarsland et al., 2010; Pedersen et al., 2017). Long-term follow-up studies demonstrate that patients with PD-MCI have a markedly increased risk of progressing to PDD. For instance, a 5-year population-based study found that approximately 39.1% of patients with early PD-MCI eventually progressed to dementia (Pedersen et al., 2017).

The progression to PDD severely compromises patients' quality of life, increases caregiver burden (Leroi et al., 2012), and is associated with elevated mortality rates (Hely et al., 2008). Furthermore, PD-MCI significantly impairs patients' capacity to manage complex daily activities, including medication adherence and financial planning. Therefore, early and accurate identification of PD-MCI is of paramount clinical importance, as it enables the development of individualized intervention strategies, implementation of supportive measures, patient and caregiver education, and enhancement of overall safety and quality of life—all while potentially delaying progression to dementia and improving long-term patient outcomes.

Given the clinical importance of early PD-MCI identification, numerous studies have attempted to develop predictive models by integrating diverse multimodal data. These modalities range from clinical and neuropsychological assessments to advanced neuroimaging, biofluid and genetic markers, electrophysiological recordings, and kinematic data (Hosseinzadeh et al., 2023; Zhu Y. et al., 2024; Amboni et al., 2022; Russo et al., 2023; Parajuli et al., 2023). However, the development of practical diagnostic models still faces substantial challenges, as highlighted in recent systematic reviews (Altham et al., 2024; Guo et al., 2021; Wu et al., 2025). One major limitation is the small cohort sizes used in many studies, which can compromise statistical power and limit the generalizability of the findings (Altham et al., 2024). Additionally, a critical methodological flaw is the common use of visit-level data splits, which can cause longitudinal information leakage. This occurs when multiple samples from the same subject are included in both training and testing datasets, leading to overly optimistic performance estimates that may not hold in real-world clinical settings (Veetil et al., 2024; Yagis et al., 2021). This issue contributes to the high risk of bias and lack of external validation frequently observed in the field (Altham et al., 2024). Furthermore, there is often limited algorithmic diversity and suboptimal hyperparameter tuning, with many studies failing to report key details about model architectures and parameters, which restricts reproducibility and the exploration of potentially more effective configurations (Altham et al., 2024).

To address these limitations, this study presents a robust framework for the classification of PD-MCI using a large-scale, multi-visit clinical dataset. The primary contributions and innovations of this work are as follows:

(1) Large-scale data analysis: we leverage the publicly available Parkinson's Progression Markers Initiative (PPMI) database, ensuring sufficient statistical power and enhancing the reliability of our findings.
(2) Methodological rigor: to prevent information leakage from multiple visits per patient, we implement a strict subject-level stratified sampling protocol for splitting data into training and testing sets, ensuring that all records from a single individual belong exclusively to one set.
(3) Systematic feature selection: we employ least absolute shrinkage and selection operator (LASSO) logistic regression to systematically identify the most predictive clinical features from a wide array of candidates, promoting model parsimony and interpretability.
(4) Optimal model comparison: we construct, compare, and rigorously evaluate four distinct machine learning algorithms: logistic regression (LR), support vector machine (SVM), random forest (RF), and extreme gradient boosting (XGBoost). We utilize subject-level stratified cross-validation with Bayesian optimization for hyperparameter tuning, ensuring optimal model performance.
(5) Advanced evaluation and interpretability: we assess model performance using multiple metrics optimized for imbalanced data and provide clinical insights through various feature importance analysis techniques (e.g., coefficients, Shapley additive explanations (SHAP), permutation importance) to ensure transparent and clinically relevant decision-making processes.

Through this structured approach, we aim to develop and validate a practical and accurate classification model that can serve as a reliable tool for clinicians in the early identification of PD-MCI, potentially improving patient care and clinical decision-making.

## 2 Materials and methods

**Figure 1:**
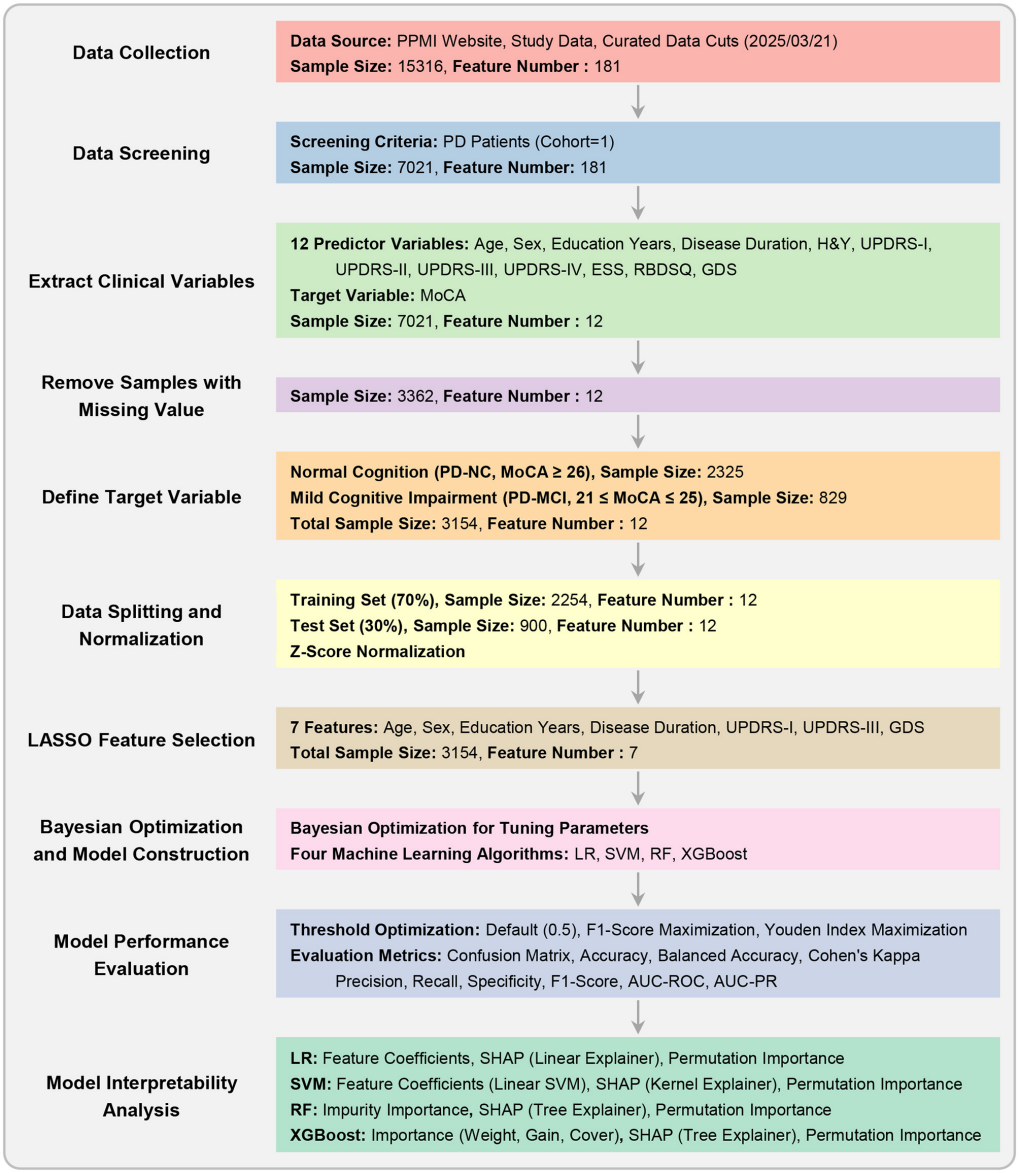
Experimental workflow. This flowchart outlines the key stages of the study, from data collection and preprocessing to model construction, hyperparameter optimization, performance evaluation, and interpretability analysis.

The overall experimental workflow is illustrated in Figure 1. The methodology was designed to ensure robust model development and validation, with a strong emphasis on preventing data leakage. Our approach encompasses the following key stages: (1) dataset preparation and quality control, (2) subject-level stratified data splitting and Z-score normalization, (3) LASSO-based feature selection, (4) model construction and hyperparameter optimization using Bayesian optimization, (5) comprehensive model evaluation with multiple threshold optimization strategies, and (6) feature importance analysis for model interpretability.

### 2.1 Dataset description

The research data were sourced from the publicly available Parkinson's Progression Markers Initiative (PPMI) database (https://www.ppmi-info.org) (Marek et al., 2011, 2018). The PPMI study was approved by the institutional review board at each participating site, and all participants provided written informed consent. Our study included only data from PD patients, resulting in a dataset containing records from multiple visits.

From this dataset, we extracted 12 potential predictor variables covering patients' demographic information, disease characteristics, and clinical assessment scores, which have been widely identified as significant predictors of cognitive decline in previous prospective cohort studies and meta-analyses (Guo et al., 2021; Hosseinzadeh et al., 2023; Schrag et al., 2017). These features, with their full names and abbreviations used hereafter, are: age at visit (age), sex, years of education (EDUCYRS), disease duration, Hoehn and Yahr stage (H&Y), Unified Parkinson's Disease Rating Scale part I (UPDRS-I), part II (UPDRS-II), part III (UPDRS-III), part IV (UPDRS-IV), Epworth Sleepiness Scale (ESS), Rapid Eye Movement Sleep Behavior Disorder Screening Questionnaire (RBDSQ), and Geriatric Depression Scale (GDS). Disease duration was calculated by subtracting the age at onset from the age at each visit. The patient identifier (PATNO) was used exclusively for subject-level data splitting.

The target variable for classification was determined based on the Montreal Cognitive Assessment (MoCA) score, a widely used screening tool for cognitive impairment in PD (Jeon et al., 2022). Following the commonly accepted cutoff of 26 for defining normal cognition (Hosseinzadeh et al., 2023), each visit was assigned to one of two classes. Class 0 (PD-NC) included patients with normal cognition, defined by a MoCA score ≥ 26. Class 1 (PD-MCI) comprised patients with mild cognitive impairment, defined by a MoCA score between 21 and 25 (inclusive). To maintain a clear distinction between the PD-MCI and more severe cognitive impairment or dementia stages, samples with MoCA scores ≤ 20 were excluded, as such scores are typically observed in patients who have already progressed to dementia (Jeon et al., 2022).

### 2.2 Data splitting

Longitudinal datasets present inherent challenges due to multiple visits per patient, creating dependencies between data points that can lead to information leakage—a critical methodological issue quantitatively demonstrated in recent analyses (Veetil et al., 2024; Yagis et al., 2021). Such leakage allows models to exploit temporal patterns and individual-specific characteristics, resulting in artificially inflated performance estimates that fail to generalize to new patients in real-world clinical settings.

To address this challenge, we implemented subject-level stratified sampling to divide the dataset into training (70%) and test (30%) sets through a strict three-stage process. First, each patient was assigned a single label based on their visits—“1” if they had at least one PD-MCI visit, “0” otherwise. The unique patient list was then stratified by this label and split accordingly. Finally, all samples from training patients were allocated to the training set, and all samples from testing patients to the test set, ensuring no patient's data appeared in both sets.

Following data splitting, all computational procedures, including feature selection, model training, and hyperparameter optimization, exclusively utilized training set information, with the test set reserved solely for final evaluation to maintain validation integrity.

### 2.3 Z-score normalization

Z-score normalization was performed using the mean and standard deviation calculated exclusively from the training data, and this transformation was subsequently applied to both the training and test sets. This approach ensures that no information from the test set influences the training process, thereby maintaining the integrity of the hold-out evaluation. Note that this normalization step was conducted only once after data splitting. Subsequent cross-validation procedures were conducted on the standardized training set, and it was not necessary to perform normalization repeatedly during the cross-validation process.

### 2.4 Feature selection using LASSO logistic regression

To identify the most critical predictors of PD-MCI from the initial 12 clinical variables, we employed least absolute shrinkage and selection operator (LASSO) logistic regression (Tibshirani, 1996). Unlike standard logistic regression, LASSO logistic regression incorporates an L1 regularization term into the cost function, which penalizes the absolute magnitude of the model's coefficients. A key advantage of this method is its ability to shrink the coefficients of less important features to exactly zero, effectively performing automatic feature selection. This is particularly suitable for a binary classification task, as it results in a more parsimonious and interpretable model by retaining only the features with the strongest predictive power.

The optimal regularization parameter λ was determined through subject-level stratified 10-fold cross-validation conducted exclusively on the training set. Following the identification of the optimal λ value, the LASSO logistic regression model was retrained on the complete training dataset using this optimized regularization parameter. Features exhibiting non-zero coefficients in the final LASSO model were identified as the selected feature subset for subsequent analysis. This carefully curated feature subset was then employed across all downstream analytical procedures, including model construction, hyperparameter optimization, and performance evaluation.

To ensure the robustness of our LASSO-based feature selection approach and assess potential methodological biases, we conducted comprehensive validation experiments detailed in the Supplementary material (Experiments II and IV). These validation analyses comprised two critical components: (1) training and evaluating all four machine learning models using the complete set of 12 initial features without any prior feature selection, thereby establishing baseline performance metrics independent of LASSO feature selection, and (2) conducting a systematic comparison of feature importance rankings across multiple selection methodologies, including filter, wrapper, and embedded approaches. This comprehensive validation framework was specifically designed to demonstrate that our primary findings and clinical conclusions remain consistent across different feature selection strategies, thereby confirming that our results are not artifacts of a single methodological approach.

### 2.5 Model construction

Using the selected feature subset from LASSO feature selection, four widely-used machine learning models (Amoroso et al., 2023; Tan et al., 2025) were constructed for PD-MCI classification: logistic regression (LR), support vector machine (SVM), random forest (RF), and extreme gradient boosting (XGBoost). LR served as the baseline linear model for binary classification, providing interpretable coefficients and establishing a foundational performance benchmark. The model employs the logistic function to map linear combinations of input features to probability estimates for PD-MCI classification (Hosmer et al., 2013). SVM was implemented with multiple kernel options to capture both linear and non-linear decision boundaries, offering flexibility in modeling complex feature relationships. The algorithm constructs optimal separating hyperplanes to distinguish between PD-MCI and PD-NC classes while maximizing the margin between classes (Cortes and Vapnik, 1995). RF, as an ensemble method utilizing multiple decision trees, was employed to reduce overfitting and improve generalization through bootstrap aggregation. This algorithm constructs numerous decision trees using random subsets of features and training samples, with final predictions determined by majority voting across all trees (Breiman, 2001). XGBoost, a gradient boosting framework, was selected for its demonstrated effectiveness in handling imbalanced datasets and its optimization capabilities for classification tasks. The algorithm sequentially builds weak learners, with each subsequent tree focusing on correcting the errors made by previous iterations (Chen and Guestrin, 2016).

To address the inherent class imbalance in the dataset, where PD-NC samples substantially outnumber PD-MCI samples, tailored strategies were implemented for each algorithm to ensure optimal classification performance. For LR, SVM, and RF, the class weight parameter was configured to “balanced,” which automatically adjusts class weights inversely proportional to their respective frequencies in the training data, thereby providing equal importance to both minority and majority classes during model training (Pedregosa et al., 2011). For XGBoost, the scale positive weight parameter, which represents the ratio of negative to positive samples, was treated as a hyperparameter to be optimized during the hyperparameter tuning process to achieve the most effective class balance handling strategy for this specific dataset and classification task (Chen and Guestrin, 2016).

### 2.6 Hyperparameter optimization

Hyperparameter tuning for each model was conducted using Bayesian optimization (Snoek et al., 2012) within a subject-level stratified 10-fold cross-validation scheme on the training set. The area under the precision-recall curve (AUC-PR) was selected as the optimization objective, which is particularly appropriate for imbalanced datasets as it emphasizes performance on the minority class. This framework ensures robust parameter tuning without overfitting while maintaining subject-level data separation integrity. The detailed hyperparameter search spaces for all algorithms are provided in the Supplementary Table S1.

After identifying the optimal hyperparameter configurations through the Bayesian optimization process, each algorithm was retrained on the complete training dataset to produce the final diagnostic classification models. This final training phase utilized the entire training set with the selected features from LASSO logistic regression and the best hyperparameter combinations determined during the optimization procedure. The resulting models represent the culmination of the systematic feature selection and hyperparameter tuning procedures, providing the most robust and optimized configurations for each algorithm. These final models were subsequently employed for comprehensive evaluation on the independent test set to assess their real-world diagnostic performance and clinical utility for PD-MCI classification.

### 2.7 Model performance evaluation

To determine the final clinical utility of each algorithm, the models equipped with their optimized hyperparameter sets were deployed for a definitive evaluation on the independent test set. The primary goal was to assess their efficacy in discriminating between PD-MCI and PD-NC individuals. Performance was quantified using a comprehensive suite of metrics, each providing unique insights into different aspects of classification performance in the clinical context of PD-MCI diagnosis. The evaluation metrics employed in this study are detailed in Table 1, which presents the mathematical formulation and clinical interpretation of each measure.

**Table 1:** Evaluation metrics for PD-MCI classification performance.

**Metric**	**Formula**	**Clinical significance in PD-MCI diagnosis**
Accuracy	$\frac{TP+TN}{TP+TN+FP+FN}$	Overall proportion of correctly classified patients, providing general diagnostic performance assessment
Balanced accuracy	$\frac{1}{2}\left(\frac{TP}{TP+FN}+\frac{TN}{TN+FP}\right)$	Accounts for class imbalance by averaging sensitivity and specificity, ensuring fair evaluation despite unequal PD-MCI and PD-NC sample sizes
Precision	$\frac{TP}{TP+FP}$	Proportion of patients classified as PD-MCI who truly have cognitive impairment, indicating diagnostic reliability and reducing false alarms
Sensitivity	$\frac{TP}{TP+FN}$	Proportion of actual PD-MCI patients correctly identified, crucial for early detection and timely intervention
Specificity	$\frac{TN}{TN+FP}$	Proportion of cognitively normal patients correctly classified, important for avoiding unnecessary anxiety and overtreatment
F1-Score	$\frac{2\times precision\times sensitivity}{precision+sensitivity}$	Harmonic mean balancing precision and sensitivity, particularly valuable for imbalanced datasets in clinical screening
Cohen's Kappa	$\frac{P_{o}-P_{e}}{1-P_{e}}$	Agreement beyond chance, accounting for random classification probability, providing robust performance assessment
AUC-ROC	Area under ROC curve	Overall discriminative ability across all threshold values, indicating model's capacity to distinguish PD-MCI from PD-NC
AUC-PR	Area under PR curve	Performance measure emphasizing positive class prediction, particularly informative for imbalanced PD-MCI classification tasks

TP, true positive; TN, true negative; FP, false positive; FN, false negative; *P*_*o*_, observed agreement; *P*_*e*_, expected agreement by chance; AUC-ROC, area under the receiver operating characteristic curve; AUC-PR, area under the precision-recall curve. Sensitivity and recall are essentially the same metric, commonly used in medical and machine learning domains, respectively.

Among these metrics, receiver operating characteristic (ROC) and PR curves are fundamental evaluation tools for binary classification performance assessment. ROC curves demonstrate a model's discriminative ability to distinguish between positive and negative classes across all classification thresholds, plotting the true positive rate (sensitivity) against the false positive rate (1-specificity). In contrast, PR curves illustrate the trade-off between precision and recall (sensitivity), providing a more informative evaluation for imbalanced datasets as they focus specifically on positive class performance and are less influenced by the large number of true negatives.

In the context of PD-MCI classification tasks, these metrics assume particular clinical significance. The inherent class imbalance in PD-MCI datasets makes AUC-PR especially informative, as it better reflects model performance on the minority class of interest. Furthermore, sensitivity and specificity are critically important in this clinical context, as they directly measure the model's ability to correctly identify patients with cognitive impairment and those who are cognitively normal, respectively. Accurate classification in this domain has profound implications for early intervention strategies and patient care management, making the comprehensive evaluation provided by both ROC and PR curve analyses essential for validating diagnostic models.

### 2.8 Threshold optimization

In clinical machine learning applications, the selection of an appropriate decision threshold is crucial for translating continuous probability outputs into binary diagnostic classifications (Freeman and Moisen, 2008). To comprehensively evaluate model performance across different clinical scenarios, we implemented three distinct thresholding strategies.

The first strategy employs the conventional default threshold of 0.5, serving as the standard baseline for binary classification. The second approach implements a threshold optimized to maximize the F1-score, which represents the harmonic mean of precision and sensitivity. This strategy is particularly well-suited for imbalanced datasets and research settings where overall diagnostic accuracy across both classes is prioritized. The third strategy employs a threshold that maximizes Youden's Index (sensitivity + specificity – 1), representing standard practice in medical diagnostics where minimizing both false positives and false negatives is crucial for optimal patient care.

For each model and thresholding strategy, threshold values were determined within the subject-level stratified 10-fold cross-validation procedure on the training set to prevent overfitting. The median of the optimal thresholds obtained across all cross-validation folds was calculated and used as the final threshold for each strategy. These final thresholds were subsequently applied to the independent test set for performance evaluation. This systematic multi-threshold comparative approach enables comprehensive understanding of each model's behavior across different clinical scenarios, allowing clinicians to select the most appropriate threshold configuration based on their specific diagnostic priorities and the relative importance of minimizing false positives vs. false negatives in their clinical setting.

### 2.9 Feature importance analysis

To enhance model interpretability and identify the clinical variables that contribute most significantly to PD-MCI prediction, we conducted comprehensive feature importance analyses for each algorithm using multiple complementary methodological approaches. The optimal models obtained from the hyperparameter optimization process were utilized directly for feature importance evaluation, ensuring methodological consistency with the model configurations employed for performance assessment. Three distinct importance measures were systematically applied: coefficient weights (Hosmer et al., 2013; Cortes and Vapnik, 1995), Shapley additive explanations (SHAP) values (Lundberg and Lee, 2017), and permutation importance (Breiman, 2001; Altmann et al., 2010).

For LR and linear SVM, three importance measures were employed. Coefficient weights were calculated as the absolute values of the learned coefficients, representing the direct linear contribution of each feature to classification decisions. SHAP explainers (linear explainer for LR and kernel explainer for SVM) were utilized to provide unified feature attribution values that satisfy efficiency and symmetry axioms. Permutation importance was computed by measuring the decrease in model performance when each feature's values are randomly shuffled.

For RF and XGBoost, both intrinsic and external importance measures were calculated. RF employed impurity-based importance by measuring the total decrease in node impurity weighted by the probability of reaching each node across all trees. XGBoost utilized the built-in gain metric, which measures the average gain across all splits using each feature. Both tree-based models were analyzed using SHAP tree explainer to provide exact feature attribution values specifically designed for ensemble methods. Permutation importance was evaluated for both algorithms by assessing the impact of feature perturbation on model performance.

This multi-faceted approach enables comprehensive understanding of feature contributions across different algorithmic paradigms and provides robust insights into the clinical variables most predictive of PD-MCI.

### 2.10 Software implementation

All analyses were implemented in Python 3.12 using scikit-learn (Pedregosa et al., 2011) for machine learning algorithms, XGBoost for gradient boosting (Chen and Guestrin, 2016), SHAP for interpretability analysis (Lundberg and Lee, 2017), and Optuna for Bayesian optimization (Akiba et al., 2019). The hyperparameter optimization employed Gaussian Process-based Bayesian optimization with expected improvement acquisition function to efficiently explore the hyperparameter space (Snoek et al., 2012). Cross-validation procedures utilized stratified sampling to maintain class distribution across folds, ensuring robust model evaluation. All experiments were conducted on a computational platform with reproducible random seeds to ensure result consistency and facilitate replication. The complete source code and implementation details are available at: https://github.com/yuzhounh/PD-MCI-Classification.

## 3 Experimental results

### 3.1 Demographic and clinical characteristics

Following preprocessing and filtering based on MoCA scores, the final dataset comprised 3,154 valid records from 896 unique patients. At the visit level, 829 records (26.28%) were classified as PD-MCI, with the remaining 2,325 classified as PD-NC. This proportion of PD-MCI is notably consistent with the 25.8% cross-sectional prevalence reported in a large multicenter study by Aarsland et al. (2010). However, when classifying at the subject level—where a patient was labeled as “1” (PD-MCI) if they had at least one visit meeting the MCI criteria—the proportion of patients classified as PD-MCI increased to 42.97%. This resulted in a cohort of 511 PD-NC and 385 PD-MCI patients for subject-level analyses.

The demographic and clinical characteristics of the study population are presented in Table 2. Statistical analysis with false discovery rate (FDR) correction revealed significant between-group differences for all variables except ESS (*p* = 0.051). The PD-MCI group was significantly older, had a higher proportion of males, fewer years of education, and showed a shorter disease duration compared to the PD-NC group (all *p* < 0.05). Clinically, the PD-MCI group exhibited significantly higher Hoehn and Yahr stage (H&Y), more severe non-motor symptoms of daily living (UPDRS-I), motor symptoms of daily living (UPDRS-II), motor signs (UPDRS-III), motor complications (UPDRS-IV), and depressive symptoms (GDS), as well as higher rates of REM sleep behavior disorder symptoms (RBDSQ) (all *p* < 0.05). In contrast, daytime sleepiness scores (ESS) showed no significant difference between groups. These findings highlight a distinct clinical and demographic profile for patients with PD-MCI, providing a strong basis for machine learning-based classification.

**Table 2:** Demographic and clinical characteristics of the study population.

**Variable**	**Range**	**Overall**	**PD-NC**	**PD-MCI**	***p*-Value**
Sample size		3,154	2,325	829	
Age	[29.74, 89.93]	65.06 ± 9.56	63.82 ± 9.44	68.53 ± 9.01	< 0.001
Sex (Male %)	{0, 1}	1 (0.64)	1 (0.62)	1 (0.69)	0.001
EDUCYRS	[0, 20]	15.77 ± 3.10	16.07 ± 2.92	14.94 ± 3.42	< 0.001
Duration	[0.15, 26.30]	6.96 ± 3.69	7.10 ± 3.76	6.57 ± 3.48	0.001
H&Y	{0–5}	2 (0.77)	2 (0.78)	2 (0.75)	0.014
UPDRS-I	[0, 35]	8.84 ± 5.61	8.50 ± 5.46	9.78 ± 5.92	< 0.001
UPDRS-II	[0, 48]	9.56 ± 6.28	9.29 ± 6.13	10.31 ± 6.63	< 0.001
UPDRS-III	[2, 96]	30.03 ± 13.75	29.54 ± 13.51	31.41 ± 14.33	0.001
UPDRS-IV	[0, 19]	2.38 ± 3.16	2.48 ± 3.21	2.12 ± 3.01	0.004
ESS	[0, 24]	7.15 ± 4.44	7.08 ± 4.46	7.36 ± 4.37	0.051
RBDSQ	[0, 13]	4.84 ± 3.24	4.76 ± 3.24	5.06 ± 3.22	0.014
GDS	[0, 15]	2.79 ± 2.87	2.57 ± 2.75	3.40 ± 3.12	< 0.001

Continuous variables are presented as mean ± standard deviation; categorical variables as mode (proportion). The *p* values were calculated using Mann–Whitney *U*-tests for continuous variables and Chi-square tests for categorical variables, with FDR correction applied.

### 3.2 Feature correlation and multicollinearity assessment

**Figure 2:**
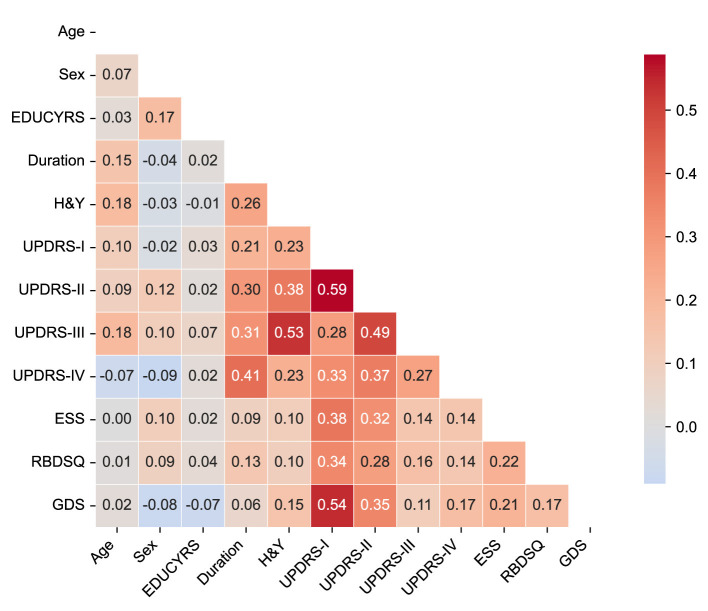
Correlation matrix of clinical features. The heatmap displays Pearson correlation coefficients between all predictor variables in the complete dataset. The color scale ranges from blue (negative correlations) to red (positive correlations). Only the lower triangular matrix is shown to avoid redundancy.

To assess potential multicollinearity among predictor variables and understand the relationships between clinical features, we examined pairwise correlations using Pearson correlation coefficients for the complete dataset. The correlation matrix is presented in Figure 2, revealing the strength and direction of associations between all predictor variables used in the classification models.

The correlation analysis demonstrated generally low to moderate correlations among most clinical features, with the highest positive correlation being *r* = 0.59 between UPDRS-I and UPDRS-II scores, and the lowest negative correlation of *r* = −0.09 between sex and UPDRS-IV. Importantly, no feature pairs exceeded the high correlation threshold of |*r*|>0.7, indicating minimal multicollinearity concerns for our machine learning models.

The strongest correlations were observed among UPDRS subscales, particularly between UPDRS-I (non-motor experiences of daily living) and UPDRS-II (motor experiences of daily living) (*r* = 0.59), and between UPDRS-II and UPDRS-III (motor examination) (*r* = 0.49). These moderate correlations reflect the expected clinical relationships within the unified rating scale framework while maintaining sufficient independence for predictive modeling. Disease duration showed meaningful positive correlations with motor severity measures, including H&Y stage (*r* = 0.26), UPDRS-II (*r* = 0.30), UPDRS-III (*r* = 0.31), and notably UPDRS-IV (motor complications) (*r* = 0.41), consistent with the progressive nature of PD. Among non-motor features, UPDRS-I demonstrated moderate associations with sleep-related measures (ESS: *r* = 0.38; RBDSQ: *r* = 0.34) and mood assessment (GDS: *r* = 0.54), reflecting the interconnected nature of non-motor symptoms in PD-MCI development.

The overall pattern of correlations supports the inclusion of all selected variables in subsequent machine learning analyses without substantial redundancy, while providing clinically interpretable relationships that align with our understanding of PD pathophysiology.

### 3.3 Feature selection results

**Figure 3:**
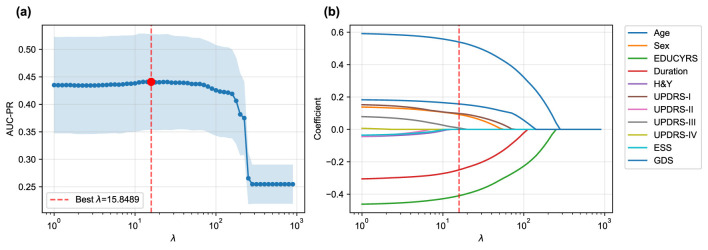
LASSO logistic regression feature selection. **(a)** Mean AUC-PR (± standard deviation) from subject-level stratified 10-fold cross-validation across a range of λ values, with the optimal λ = 15.8489 indicated. **(b)** Coefficient paths for each feature as a function of λ obtained by retraining on the complete training set. As λ increases, coefficients of less important features are progressively shrunk to zero. Only eleven features are shown as the coefficients of RBDSQ remained zero throughout the regularization path.

The LASSO logistic regression process, optimized via subject-level stratified 10-fold cross-validation on the training set to maximize AUC-PR, was used to identify the most salient predictors from the initial 12 features. Figure 3 illustrates both the performance curve derived from cross-validation and the coefficient paths obtained by retraining the model on the complete training set across a range of regularization parameters.

The cross-validation procedure identified an optimal regularization parameter of λ = 15.8489, which maximized the mean AUC-PR across all folds. At this optimal regularization strength, the LASSO algorithm selected a parsimonious subset of seven key features while shrinking the coefficients of the remaining five features (H&Y, UPDRS-II, UPDRS-IV, ESS, and RBDSQ) to zero, effectively excluding them from the final model.

While seemingly counterintuitive, the exclusion of these variables commonly associated with Parkinson's disease offers insights into the specific drivers of cognitive decline within this dataset. The exclusion of H&Y and UPDRS-II suggests that once more direct motor examination findings (UPDRS-III) and overarching demographic factors (like age and years of education) are considered, these measures of disease stage and motor disability may offer redundant or less potent predictive information for the specific task of classifying cognitive status. Similarly, while sleep disturbances measured by ESS and RBDSQ are known non-motor symptoms of PD, their predictive signal may have been overshadowed by the Geriatric Depression Scale (GDS). Given the strong interplay between depression, apathy, and cognitive function, it is plausible that GDS serves as a more powerful and direct proxy for the underlying neurobiological changes that impact cognition, thereby diminishing the independent contribution of the sleep-related features in the final LASSO model.

**Figure 4:**
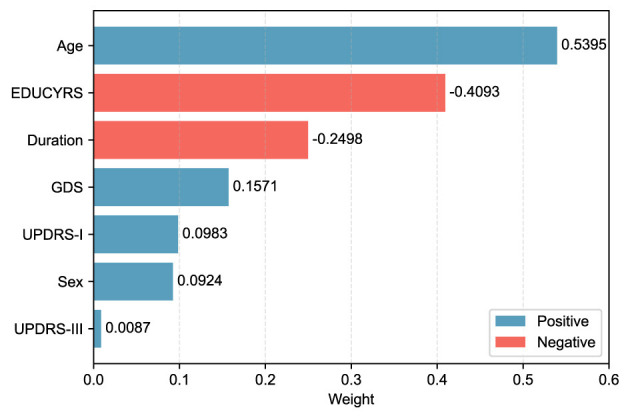
LASSO feature weights for PD-MCI classification. The horizontal bar chart displays the absolute weights of the seven selected features from the LASSO model trained on the complete training set with the optimal λ value. Blue bars represent positive coefficients (features associated with increased PD-MCI likelihood, i.e., risk features), while red bars represent negative coefficients (protective features). Features are ordered by their absolute weight magnitude, with age (0.5395) being the most influential predictor, followed by EDUCYRS (–0.4093) and Duration (–0.2498).

When the final LASSO model was trained on the complete training set using λ = 15.8489, the selected features demonstrated varying contributions to PD-MCI classification. The selected features, ranked by the absolute magnitude of their coefficients, are visualized in Figure 4. Age emerged as the most influential predictor with a positive coefficient of 0.5395, indicating that older patients have substantially higher odds of developing PD-MCI. Years of education (EDUCYRS) showed the second-largest magnitude but with a negative coefficient of –0.4093, confirming its protective role against cognitive decline. Disease duration exhibited a negative coefficient of –0.2498, suggesting that longer disease duration may be associated with better cognitive preservation in this cohort. Among the clinical severity measures, depressive symptoms (GDS) demonstrated a positive coefficient of 0.1571, while non-motor experiences of daily living (UPDRS-I) and sex showed smaller positive contributions of 0.0983 and 0.0924, respectively. Motor examination scores (UPDRS-III) had the smallest coefficient of 0.0087, indicating minimal direct contribution to the classification decision. These seven features were used for the construction and comparison of all subsequent machine learning models.

### 3.4 Hyperparameter optimization

To ensure optimal model performance prior to final testing, all machine learning algorithms underwent systematic hyperparameter optimization on the training set using subject-level stratified 10-fold cross-validation. Hyperparameters for each algorithm were optimized using Bayesian optimization with Optuna, where AUC-PR was used as the objective function to identify the optimal parameter configurations that maximize predictive performance while maintaining generalizability. The resulting optimal hyperparameters for each model are summarized in the Supplementary Table S2.

The hyperparameter optimization revealed distinct characteristics of the models that provide insights into the underlying structure of the classification problem. For LR, the optimal parameter configuration favored an L2 penalty (Ridge regularization), indicating that modest regularization was beneficial for preventing overfitting while maintaining model stability. SVM achieved optimal performance with a linear kernel, demonstrating that the decision boundary between PD-MCI and PD-NC classes can be effectively captured through linear separation in the feature space. These findings collectively suggest that the relationship between the selected clinical features and the classification target is largely linear in nature.

For the tree-based models, both RF and XGBoost favored conservative parameters designed to prevent overfitting, reflecting the complexity constraints of the dataset. RF optimization selected a moderate maximum tree depth and relatively high minimum samples per leaf, indicating that shallow, well-regularized trees were most effective for generalization. Similarly, XGBoost's optimal configuration included a maximum tree depth of only 2, further reinforcing that effective classification could be achieved with relatively simple decision rules rather than complex tree structures. The preference for conservative tree parameters across both ensemble methods suggests that the underlying patterns in the clinical data are sufficiently captured by simple decision boundaries, consistent with the linear nature of the classification problem identified through the linear model optimization results.

### 3.5 Cross-validated performance on training data

Following hyperparameter optimization, each model was evaluated on the training set using subject-level stratified 10-fold cross-validation with the obtained optimal parameters to assess their intrinsic discriminative capacity. The evaluation initially computed threshold-independent metrics (AUC-ROC and AUC-PR) that assess the model's fundamental ability to distinguish between PD-MCI and non-MCI cases across all possible decision thresholds. Subsequently, three different threshold optimization strategies were systematically applied to determine optimal decision boundaries: the default threshold (0.5), F1-score maximization, and Youden index maximization. These threshold optimization strategies specifically influence threshold-dependent metrics such as accuracy, precision, sensitivity, and F1-score. The comprehensive cross-validation performance results using the optimized hyperparameters across all threshold strategies are presented in Table 3.

**Table 3:** Cross-validation performance comparison on training data across different threshold strategies.

**Threshold**	**Metric**	**LR**	**SVM**	**RF**	**XGBoost**
	AUC-ROC	0.6948 ± 0.0441	0.6946 ± 0.0453	0.6952 ± 0.0427	**0.7076** **±0.0442**
	AUC-PR	0.4443 ± 0.0843	0.4462 ± 0.0850	0.4408 ± 0.0833	**0.4529** **±0.0807**
Default (0.5)	Accuracy	0.6406 ± 0.0427	**0.7507** **±0.0355**	0.6252 ± 0.0390	0.7098 ± 0.0415
	Balanced accuracy	**0.6487** **±0.0435**	0.5548 ± 0.0411	0.6219 ± 0.0382	0.6142 ± 0.0500
	Precision	0.3833 ± 0.0740	**0.5073** **±0.2208**	0.3616 ± 0.0619	0.4300 ± 0.1048
	Sensitivity	**0.6633** **±0.0835**	0.1534 ± 0.0840	0.6145 ± 0.0858	0.4174 ± 0.0858
	Specificity	0.6340 ± 0.0547	**0.9562** **±0.0212**	0.6293 ± 0.0580	0.8110 ± 0.0433
	F1-score	**0.4820** **±0.0702**	0.2300 ± 0.1174	0.4520 ± 0.0589	0.4192 ± 0.0839
	Cohen's Kappa	**0.2393** **±0.0806**	0.1393 ± 0.1042	0.1969 ± 0.0687	0.2280 ± 0.1050
F1-Score	Optimal threshold	0.4636 ± 0.0474	0.2217 ± 0.0467	0.4372 ± 0.1239	0.3570 ± 0.1099
	Accuracy	0.6015 ± 0.0701	0.6008 ± 0.0644	0.6203 ± 0.1138	**0.6474** **±0.1051**
	Balanced accuracy	0.6723 ± 0.0366	0.6711 ± 0.0366	0.6673 ± 0.0294	**0.6836** **±0.0475**
	Precision	0.3781 ± 0.0757	0.3762 ± 0.0723	0.4028 ± 0.0920	**0.4201** **±0.1038**
	Sensitivity	**0.8145** **±0.0812**	0.8113 ± 0.0850	0.7705 ± 0.1860	0.7615 ± 0.1187
	Specificity	0.5301 ± 0.1184	0.5309 ± 0.1089	0.5640 ± 0.2152	**0.6058** **±0.1880**
	F1-score	0.5100 ± 0.0615	0.5078 ± 0.0592	0.5092 ± 0.0520	**0.5278** **±0.0630**
	Cohen's Kappa	0.2511 ± 0.0819	0.2483 ± 0.0764	0.2620 ± 0.0879	**0.2937** **±0.1140**
Youden Index	Optimal threshold	0.4784 ± 0.0253	0.2441 ± 0.0496	0.4495 ± 0.1044	0.3643 ± 0.0770
	Accuracy	0.6187 ± 0.0620	0.6304 ± 0.0614	0.6262 ± 0.0887	**0.6528** **±0.0830**
	Balanced accuracy	0.6732 ± 0.0357	0.6721 ± 0.0354	0.6692 ± 0.0282	**0.6859** **±0.0452**
	Precision	0.3865 ± 0.0785	0.3920 ± 0.0707	0.4001 ± 0.0952	**0.4166** **±0.0966**
	Sensitivity	**0.7765** **±0.0881**	0.7512 ± 0.1314	0.7540 ± 0.1439	0.7539 ± 0.0738
	Specificity	0.5700 ± 0.0979	0.5930 ± 0.1097	0.5844 ± 0.1670	**0.6179** **±0.1368**
	F1-score	0.5080 ± 0.0612	0.5052 ± 0.0594	0.5067 ± 0.0536	**0.5276** **±0.0629**
	Cohen's Kappa	0.2595 ± 0.0783	0.2625 ± 0.0673	0.2628 ± 0.0825	**0.2950** **±0.1051**

Values are presented as mean ± standard deviation across 10-fold cross-validation. Bold values indicate the best performance for each metric across the four models.

The comprehensive cross-validation analysis revealed distinct performance patterns across the four machine learning algorithms under three threshold optimization strategies. Regarding threshold-independent metrics, XGBoost demonstrated superior discriminative capability, achieving the highest AUC-ROC and AUC-PR scores. Nevertheless, the performance differences among all algorithms were relatively modest, with all models achieving similar AUC-ROC and AUC-PR ranges, suggesting comparable inherent discriminative capacity across models for PD-MCI classification tasks.

The default threshold (0.5) strategy revealed substantial performance variations across algorithms. SVM achieved the highest accuracy and specificity, but demonstrated markedly poor sensitivity, resulting in the lowest F1-score and Cohen's kappa. This pattern indicates that the default threshold is excessively conservative for SVM in PD-MCI detection, leading to substantial underdiagnosis. In contrast, LR with the default threshold achieved more balanced performance with notably higher sensitivity, F1-score, and Cohen's kappa.

Both optimized threshold strategies demonstrated superior balance between sensitivity and specificity compared to the default threshold. The F1-score optimization strategy consistently improved sensitivity across all models while maintaining reasonable precision. XGBoost achieved the best overall performance under F1-score optimization with the highest scores across most key metrics including accuracy, balanced accuracy, precision, and Cohen's kappa. LR exhibited the highest sensitivity under this strategy. The Youden index optimization provided a similar balanced performance profile to F1-score optimization. XGBoost again demonstrated the strongest performance across most metrics, while LR maintained the highest sensitivity under Youden index optimization.

The optimal thresholds derived from cross-validation varied substantially across algorithms and optimization criteria. SVM consistently required the lowest thresholds, reflecting its tendency to produce conservative probability estimates. XGBoost required intermediate thresholds, while LR and RF showed higher and more variable threshold requirements.

Based on these comprehensive cross-validation results, XGBoost emerged as the most promising algorithm across both optimized threshold strategies, consistently achieving the highest F1-scores and Cohen's kappa values. The optimized threshold strategies (F1-score and Youden index) demonstrated clear superiority over the default threshold for PD-MCI classification, providing more clinically relevant sensitivity-specificity trade-offs. For subsequent test set evaluation, the median optimized thresholds from cross-validation were adopted to ensure robust and generalizable performance estimates.

### 3.6 Model evaluation

**Figure 5:**
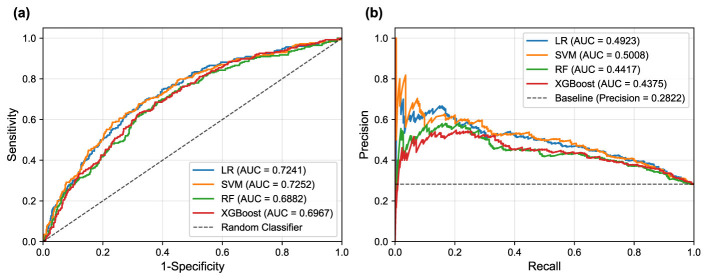
ROC and PR curves for the different machine learning models on the test set. **(a)** ROC curves illustrate the trade-off between sensitivity and 1-specificity. **(b)** PR curves show the trade-off between precision and recall (sensitivity).

The performance of the four machine learning models was evaluated on the independent test set using the median optimized thresholds derived from cross-validation. Figure 5 illustrates the corresponding ROC and PR curves for all models, providing visual representation of their discriminative performance. Table 4 presents a detailed comparison of model performance across different threshold strategies, providing both threshold-independent metrics (AUC-ROC and AUC-PR) and threshold-dependent metrics under various optimization criteria. The comprehensive evaluation of the four models revealed important insights into their discriminative abilities and the critical role of threshold optimization in imbalanced classification scenarios.

**Table 4:** Performance comparison on test data across different threshold strategies.

**Threshold**	**Metric**	**LR**	**SVM**	**RF**	**XGBoost**
	AUC-ROC	0.7241	**0.7252**	0.6882	0.6967
	AUC-PR	0.4923	**0.5008**	0.4417	0.4375
Default (0.5)	Accuracy	0.6689	**0.7356**	0.6344	0.6889
	Balanced accuracy	**0.6774**	0.5614	0.6522	0.6256
	Precision	0.4447	**0.6212**	0.4122	0.4519
	Sensitivity	**0.6969**	0.1614	0.6929	0.4803
	Specificity	0.6579	**0.9613**	0.6115	0.7709
	F1-score	**0.5429**	0.2563	0.5169	0.4656
	Cohen's Kappa	**0.3027**	0.1583	0.2522	0.2465
F1-Score	Optimal threshold	0.4769	0.2452	0.4465	0.3942
	Accuracy	0.6244	**0.6300**	0.5578	0.6222
	Balanced accuracy	**0.6667**	0.6634	0.6358	0.6449
	Precision	0.4110	**0.4132**	0.3710	0.4023
	Sensitivity	0.7638	0.7402	**0.8150**	0.6969
	Specificity	0.5697	0.5867	0.4567	**0.5929**
	F1-score	**0.5344**	0.5303	0.5099	0.5101
	Cohen's Kappa	**0.2645**	0.2636	0.1993	0.2371
Youden Index	Optimal threshold	0.4840	0.2495	0.4629	0.3767
	Accuracy	**0.6433**	0.6356	0.5722	0.6111
	Balanced accuracy	**0.6751**	0.6649	0.6351	0.6431
	Precision	**0.4251**	0.4170	0.3757	0.3957
	Sensitivity	0.7480	0.7323	**0.7795**	0.7165
	Specificity	**0.6022**	0.5975	0.4907	0.5696
	F1-score	**0.5421**	0.5314	0.5070	0.5098
	Cohen's Kappa	**0.2846**	0.2683	0.2038	0.2297

Bold values indicate the best performance for each metric across the four models.

In terms of overall discriminative ability, SVM demonstrated superior performance, achieving the highest AUC-ROC and AUC-PR scores. LR followed closely with comparable AUC scores, indicating robust classification potential. These results suggest that linear models possess excellent discriminative power for PD-MCI classification in this dataset, likely due to their ability to capture the linear relationships between the selected clinical features and cognitive impairment status. While showing respectable performance, RF and XGBoost achieved lower AUC values, suggesting that the additional complexity of ensemble methods may not provide substantial benefits for this particular feature set and dataset.

The default threshold of 0.5 again proved suboptimal, as exemplified by the SVM's performance: while achieving high specificity and precision, its sensitivity was extremely low, resulting in an unacceptably low F1-score. Such performance characteristics would be problematic in clinical scenarios where high sensitivity is crucial for detecting cognitive impairment, as missing PD-MCI cases could delay appropriate interventions and patient care planning. This underscores the fundamental necessity of threshold optimization when dealing with imbalanced datasets to achieve an effective trade-off between sensitivity and specificity that aligns with clinical priorities.

The two threshold optimization strategies, i.e., maximizing the F1-score and maximizing the Youden Index, yielded substantially more balanced performance across all models. Under F1-score optimization, the LR model demonstrated superior performance across the majority of evaluation metrics, achieving the highest balanced accuracy, F1-score, and Cohen's Kappa. Similarly, under Youden Index optimization, the LR model again secured the best performance in most metrics. The SVM model consistently achieved competitive performance under both optimization strategies, particularly showing strong results in F1-score optimization. This consistent performance highlights the strength of both linear models, particularly the LR model, in achieving well-rounded and balanced overall performance for PD-MCI classification. The LR model's interpretability, combined with its robust performance, makes it particularly suitable for clinical applications where understanding the contribution of individual features is important for clinical decision-making.

However, two notable exceptions emerged from the threshold optimization results that merit careful consideration. When the threshold was optimized to maximize the F1-score, RF achieved the highest sensitivity, while under Youden Index optimization, the RF model secured the top performance in sensitivity again. These findings indicate that if the primary clinical objective is to identify the maximum number of PD-MCI cases (i.e., maximizing sensitivity to minimize missed diagnoses), appropriately optimized RF models might be more suitable choices than linear models. The RF model's ability to achieve high sensitivity values suggests that for clinical applications where the cost of false negatives is particularly high—such as screening scenarios where missing cognitive impairment could lead to delayed treatment—ensemble models with optimized thresholds could be preferred despite their lower overall discriminative ability.

These findings highlight the fundamental importance of aligning model selection and threshold optimization with specific clinical objectives. For applications prioritizing the minimization of false positives (high specificity) or seeking the best overall diagnostic accuracy, LR and SVM demonstrate superior performance. Conversely, for scenarios where maximizing the detection of PD-MCI patients is paramount, RF models with appropriately optimized thresholds may provide better clinical utility despite potentially higher false positive rates.

### 3.7 Feature importance analysis

To gain deeper insights into the decision-making processes of our models and identify the most influential clinical factors for PD-MCI classification, we conducted a comprehensive feature importance analysis after training each of the four models (LR, SVM, RF, XGBoost) on the complete training dataset using the seven selected features and optimized hyperparameters. We employed multiple complementary analytical approaches to ensure robust and comprehensive assessment: model-specific importance measures (coefficients for linear models, impurity-based scores for RF, and Gain for XGBoost), SHAP values for understanding individual feature contributions, and the model-agnostic permutation importance method to corroborate our findings from multiple perspectives.

**Figure 6:**
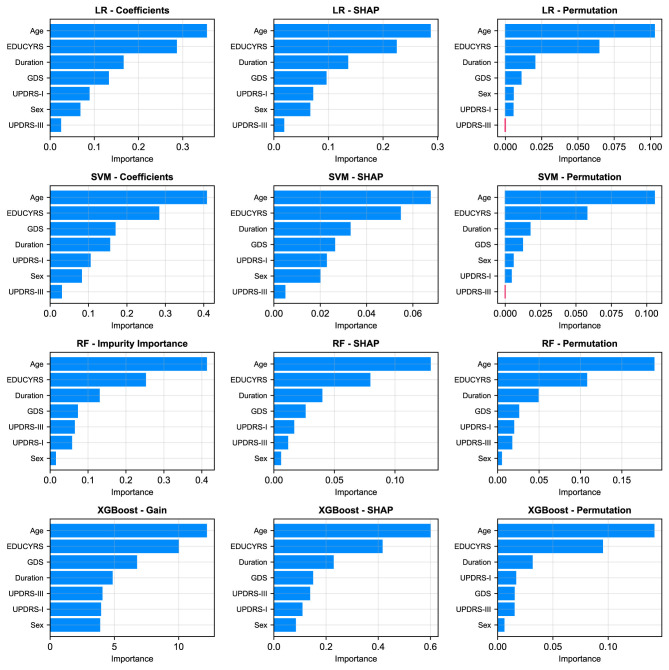
Comparison of feature importance across four models (rows: LR, SVM, RF, XGBoost) using three different evaluation metrics (columns). The metrics are: model-specific importance (coefficients for LR/SVM, Gini Impurity for RF, and Gain for XGBoost), mean absolute SHAP values, and permutation importance.

As illustrated in Figure 6, a remarkably uniform pattern emerges across all models and analytical methodologies. Three clinical variables consistently rank as the most salient predictors of cognitive status: age, years of education, and disease duration, which respectively reflect the natural progression of cognitive decline, cognitive reserve capacity, and cumulative pathological burden. Additionally, GDS frequently appears among the top four important features, underscoring the significant relationship between depressive symptoms and PD-MCI. Although each of the seven selected features contributes to overall model performance, UPDRS-III (motor examination scores) and sex exhibited consistently lower importance rankings across multiple models and evaluation metrics. This finding indicates that while these features retain predictive value, their direct contribution to PD-MCI classification is more modest relative to the predominant demographic, educational, and mood-related variables.

**Figure 7:**
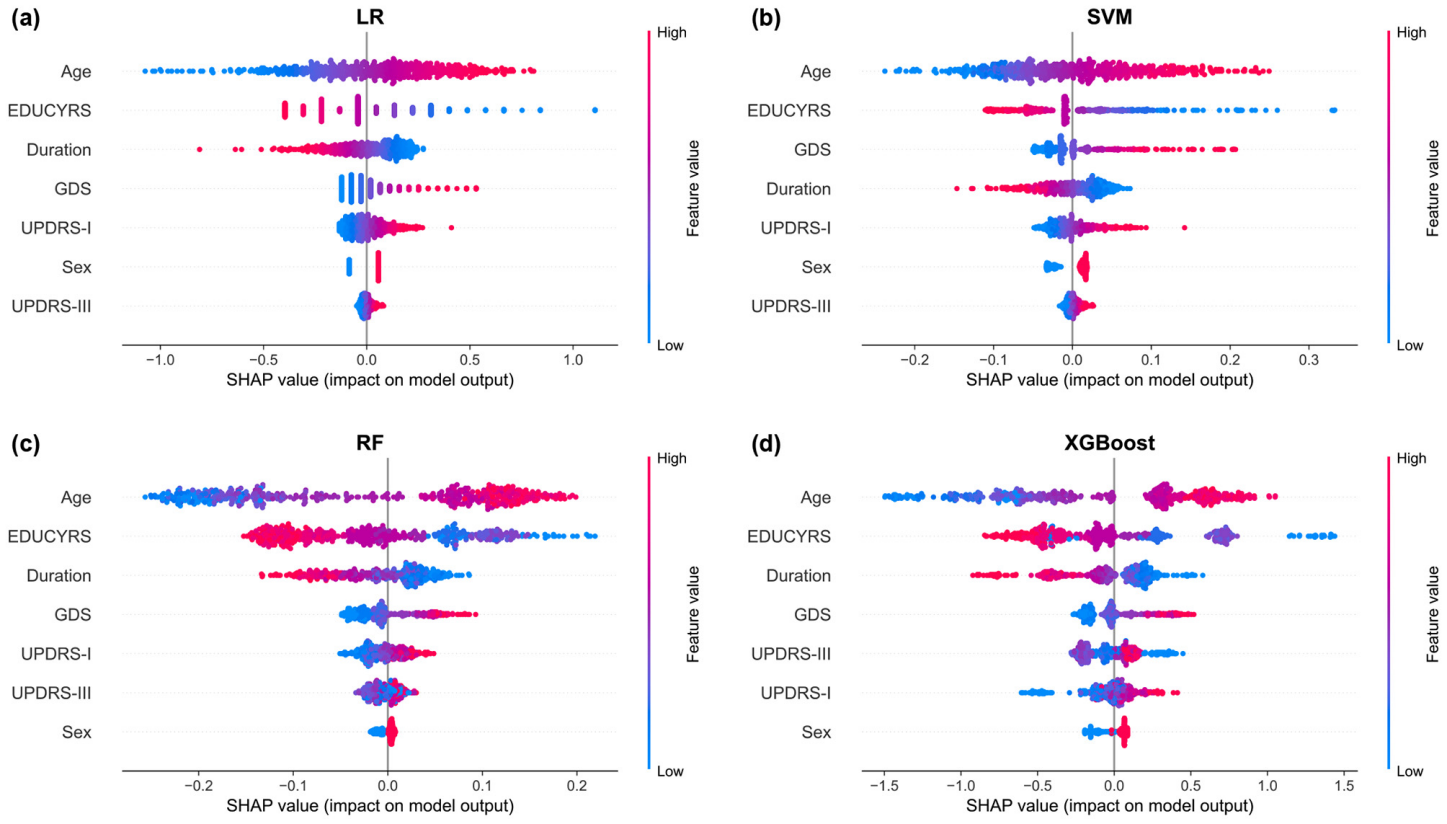
SHAP summary plots for **(a)** LR, **(b)** SVM, **(c)** RF, and **(d)** XGBoost models. Each point represents a single observation. The horizontal position indicates the feature's impact on the model output (SHAP value), while the color denotes the feature's magnitude (red for high, blue for low). Features are ranked vertically by their global importance, providing a detailed view of both the direction and consistency of their effects.

The SHAP summary plots, presented in Figure 7, provide granular insights into both the magnitude and directionality of each feature's contribution to model predictions. These visualizations reveal that higher values for age and GDS (represented by red points) are consistently associated with positive SHAP values, indicating an increased probability of PD-MCI classification (Guo et al., 2021; Schrag et al., 2017). Conversely, higher EDUCYRS values are associated with negative SHAP values, demonstrating the protective effect of education against cognitive decline. This pattern aligns with established neurological literature suggesting that educational attainment may contribute to cognitive reserve, potentially delaying the onset or manifestation of cognitive impairment in neurodegenerative diseases (Nie et al., 2019; Galtier et al., 2016).

## 4 Discussion

### 4.1 Principal findings

In this study, we systematically developed and validated machine learning models for the classification of PD-MCI using a large-scale clinical dataset from the PPMI. Our principal findings demonstrate that a parsimonious set of seven clinical features can effectively distinguish between PD patients with and without MCI. The linear models, SVM and LR, demonstrated superior overall discriminative ability with AUC-ROC scores of 0.7252 and 0.7241, respectively, accompanied by AUC-PR values of 0.5008 and 0.4923, respectively. The feature importance analysis revealed consistent patterns across all models and analytical methodologies, with age, years of education, and disease duration emerging as the most salient predictors of cognitive status.

A critical observation from our results is the severe performance imbalance exhibited by the SVM model when using the default 0.5 decision threshold, where it achieved exceptionally high specificity (0.9613) but clinically unacceptable sensitivity (0.1614). This pattern exemplifies a classic pitfall in which standard machine learning defaults prove inadequate for imbalanced clinical datasets. The model exhibited a pronounced bias toward the majority class (PD-NC), consequently failing to identify the vast majority of PD-MCI cases. Importantly, this finding does not reflect an inherent failure of the SVM algorithm but rather highlights the fundamental inadequacy of applying fixed, arbitrary thresholds in clinical contexts. This observation strongly reinforces the rationale underlying our study's emphasis on threshold optimization. By systematically adjusting the decision threshold to maximize clinically relevant metrics like the F1-score or Youden's Index, we successfully recalibrated the SVM model to achieve substantially more balanced and clinically meaningful performance, with marked improvements in sensitivity while maintaining reasonable specificity levels.

### 4.2 Comparison with prior work

Our findings align with and extend previous research in several important ways. The critical role of age, years of education, and disease duration as key predictors confirms established risk factors identified in prior studies (Guo et al., 2021; Schrag et al., 2017; Sollinger et al., 2010; Nie et al., 2019). Specifically, age has been consistently identified as a primary risk factor for cognitive decline in PD, with older patients demonstrating significantly higher rates of cognitive impairment progression (Hely et al., 2008). Similarly, educational attainment has been recognized as a protective factor, with higher education levels associated with delayed onset of cognitive symptoms, likely through enhanced cognitive reserve mechanisms (Stern, 2002; Monastero et al., 2018; Galtier et al., 2016; Nie et al., 2019). Disease duration, while presenting complex relationships in longitudinal cohorts, has been established as a fundamental predictor of cognitive deterioration in multiple prospective studies (Guo et al., 2021; Schrag et al., 2017).

However, our work distinguishes itself through two key advantages: the utilization of a large-scale dataset and rigorous methodological approaches. First, our study leverages the comprehensive PPMI database (Marek et al., 2011, 2018), which provides a substantially larger sample size compared to most previous investigations in this field (Altham et al., 2024), thereby enhancing the statistical power and generalizability of our findings. Second, we employed methodologically rigorous approaches, particularly the use of subject-level data splitting to ensure realistic performance estimates and avoid the inflated results that can arise from data leakage in longitudinal datasets (Veetil et al., 2024; Yagis et al., 2021). Additionally, our comprehensive feature selection methodology, extensive hyperparameter optimization, and multifaceted model evaluation framework contribute to the methodological rigor of this investigation (Altham et al., 2024; Wu et al., 2025).

While our purely clinical model achieved a moderate AUC-ROC of 0.7252, this performance should be evaluated within the context of its intended application as an accessible screening tool. This level of discriminative ability, derived exclusively from readily available clinical variables, represents meaningful performance for initial risk stratification and early detection purposes, though it remains insufficient for standalone definitive diagnosis.

Our results inevitably fall below the performance benchmarks established by studies incorporating complex multimodal approaches—including neuroimaging, biofluid markers, and kinematic analysis—which have reported AUC-ROC values ranging from 0.79 to 0.84 (Zhu Y. et al., 2024; Hosseinzadeh et al., 2023; Amboni et al., 2022). However, this performance gap reflects a fundamental trade-off between diagnostic accuracy and practical accessibility. While complex multimodal models offer superior discriminative performance, our approach provides a highly scalable and immediately implementable solution that can be deployed across diverse clinical settings without specialized equipment or expertise. This contribution aligns with the growing emphasis on machine learning approaches in PD-MCI detection highlighted in recent systematic reviews (Altham et al., 2024; Wu et al., 2025), demonstrating that methodologically rigorous analysis of clinical data with sufficient sample size can achieve clinically meaningful screening performance for routine healthcare applications.

### 4.3 Robustness of findings

A major potential limitation of this study is the reliance on a single cohort (PPMI). To evaluate the generalizability of our models in the absence of independent external datasets, we conducted a rigorous “pseudo-external” validation by splitting the dataset at the clinical site level, ensuring that training and testing data originated from completely different medical institutions (detailed in Supplementary Experiment I). Under this more challenging validation scenario, model performance showed modest decreases but maintained meaningful discriminative ability across sites. Remarkably, the LR model demonstrated enhanced performance on the critical precision-recall metric, with AUC-PR improving from 0.4923 to 0.5134, providing compelling evidence for its robust cross-site generalizability. The consistent performance across diverse clinical sites, despite the more stringent evaluation conditions, strengthens confidence in the real-world applicability of our approach.

Cross-methodological validation analyses rigorously confirmed the robustness of our LASSO-selected seven-feature subset (detailed in Supplementary Experiments II, IV). When evaluated using the complete 12-feature set, three core predictors—age, years of education, and disease duration—consistently emerged as the most critical variables across diverse models and importance metrics. This finding was further reinforced through systematic comparison across different feature selection families (filter, wrapper, and embedded methods), which demonstrated remarkable consensus in identifying these three variables as the most dominant predictors. Crucially, both supplementary validation experiments showed perfect concordance with our primary LASSO-selected seven-feature subset, providing compelling evidence that these features represent genuine predictive relationships rather than methodological artifacts. The convergence of findings across multiple independent analytical frameworks establishes strong confidence in the clinical relevance and diagnostic utility of our identified predictors, supporting their potential for practical implementation in PD-MCI screening.

Feature importance analysis indicated that UPDRS-III and sex consistently showed lower relative importance compared to the other five features across the majority of models and analytical approaches. To explore whether a more streamlined model could sustain similar performance, we conducted a systematic ablation study by excluding these two less significant features (detailed in Supplementary Experiments III). However, the results revealed a significant decline in the performance of LR and SVM. Therefore, these two relatively less important features are necessary for maximizing model performance.

### 4.4 Clinical implications

Our findings have direct relevance for routine clinical practice across multiple dimensions. The robust performance achieved using only readily available clinical data provides compelling evidence for the utility of these models as accessible, low-cost, and non-invasive first-line screening tools. This advantage is particularly valuable in clinical settings where advanced neuroimaging or biomarker testing may not be readily available—a critical need highlighted by recent reviews and expert consensus reports calling for practical, scalable tools to address unmet needs in PD cognitive care and facilitate implementation of predictive models into routine practice (Goldman et al., 2018b; Altham et al., 2024).

#### 4.4.1 Threshold optimization and clinical trade-offs

The threshold optimization results reveal crucial insights regarding the inherent trade-offs between performance metrics in PD-MCI classification. When optimized to maximize F1-score, the RF model achieved the highest sensitivity of 0.8150, successfully identifying over 80% of all cognitive impairment cases. Under Youden Index optimization, the RF model maintained exceptional sensitivity (0.7795), demonstrating consistent capability in capturing a substantial proportion of PD-MCI patients across different optimization strategies.

These consistently high sensitivity values carry profound clinical significance. The primary objective of a screening tool is typically to minimize false negatives (missed diagnoses), even at the cost of higher false positive rates—a principle underlying many clinical diagnostic criteria (Postuma et al., 2015; Litvan et al., 2012). From a clinical perspective, such high sensitivity translates to enhanced screening effectiveness and improved patient outcomes, ensuring that the vast majority of patients with cognitive impairment are identified and can receive timely neuropsychological assessment and appropriate therapeutic intervention.

However, high sensitivity must be balanced against increased false positive rates, which could lead to unnecessary patient anxiety and additional healthcare resource utilization for confirmatory testing. The optimal choice between linear models (offering better overall discriminative ability) and ensemble models (providing higher sensitivity) should be guided by specific clinical context, available resources, and the relative costs of false negatives vs. false positives in the particular healthcare setting.

#### 4.4.2 Clinical decision framework

To facilitate clinical decision-making, Table 5 provides a practical framework for model selection based on specific clinical scenarios:

**Table 5:** Clinical decision framework for model selection in PD-MCI classification.

**Clinical scenario**	**Primary objective**	**Model**
Screening & Early detection	Maximize case identification	RF
Research & Cohort studies	Comprehensive cognitive phenotyping	RF
Precision diagnostic assistance	Balanced accuracy & overall discrimination	LR or SVM
Resource-limited settings	Cost-effective, interpretable screening	LR

Screening and early detection programs: for settings prioritizing identification of as many PD-MCI cases as possible (a “better safe than sorry” approach), RF with optimized thresholds is recommended due to superior sensitivity performance (0.8150 under F1-score optimization). This approach is particularly valuable in primary care settings, specialty movement disorder clinics conducting routine cognitive assessments, and research cohorts requiring comprehensive cognitive phenotyping.

Precision diagnostic assistance: for contexts where balanced accuracy and overall discriminative ability are prioritized, LR and SVM are more suitable, offering superior AUC-ROC performance (0.7241 and 0.7252, respectively) and better balance across multiple evaluation metrics. This approach is appropriate for confirmatory diagnostic processes, specialist referral decisions, and clinical contexts where false positives carry significant consequences for patient care or resource allocation.

Resource-limited settings: where cost-effectiveness and interpretability are paramount, the LR model represents the optimal choice. It provides transparent decision-making processes that clinicians can easily understand and implement while maintaining sufficient predictive accuracy for practical screening applications without requiring sophisticated computational infrastructure.

This stratified approach aligns with personalized medicine goals, where predictive tools are tailored to specific clinical needs, from broad-based screening to targeted diagnostic support (Guo et al., 2021).

#### 4.4.3 Model transparency and clinical trust

The SHAP analysis enhances model transparency by illustrating the specific impact of individual factors on predictions, which can increase clinical trust and aid in patient communication. By clearly visualizing how factors like higher age increase MCI risk while more years of education decrease it, the analysis provides valuable insights for shared decision-making processes and patient education about modifiable and non-modifiable risk factors influencing cognitive health trajectory. Such interpretability is particularly valuable, as model transparency is considered a key factor for fostering clinical trust and facilitating adoption of machine learning tools in healthcare (Amann et al., 2020).

### 4.5 Strengths and limitations

This study demonstrates several key strengths that enhance the reliability and clinical applicability of our findings. First, we utilized a large-scale, high-quality dataset from the PPMI with standardized assessment protocols and rigorous quality control measures, providing a robust foundation for model development. Second, our methodological framework incorporated subject-level data splitting to prevent information leakage, comprehensive feature selection using multiple complementary techniques, and extensive hyperparameter optimization to ensure reliable model performance. Third, we conducted systematic feature importance analysis through multiple approaches—including model-specific measures, SHAP values, and permutation importance—to provide comprehensive insights into model decision-making processes. Finally, our focus on readily available clinical features enhances practical implementation feasibility across diverse clinical settings.

Several important limitations warrant consideration. First, our analysis relied exclusively on clinical data which, while accessible and cost-effective, may not fully capture the complexity of PD-MCI progression. Second, we observed an unexpected finding where disease duration appeared protective against cognitive impairment—contradicting established clinical knowledge that longer disease duration typically increases MCI risk. This counterintuitive result likely reflects selection bias inherent in longitudinal cohort designs, where patients with rapid cognitive decline may develop MCI early and subsequently withdraw from long-term studies, while those maintaining cognitive function despite longer disease duration represent a selected population of “cognitive survivors.” Third, our two-stage methodological approach performed LASSO feature selection followed by Bayesian optimization on the selected features, both using subject-level stratified 10-fold cross-validation. While nesting LASSO within the Bayesian optimization process might theoretically improve generalization performance, such complexity would substantially compromise model interpretability and practical implementation. Our current pipeline effectively mitigates information leakage while maintaining clinical utility through rigorous cross-validation procedures. Fourth, this study addressed longitudinal data through subject-level splitting to effectively prevent data leakage, but did not model the trajectory of cognitive changes over time. Future research could employ longitudinal data analysis methods such as mixed-effects models or survival analysis to explore the dynamic processes of disease progression more comprehensively. Finally, despite the PPMI dataset's high quality, external validation across truely independent datasets from diverse populations, ethnic groups, and healthcare systems remains essential to establish broader generalizability.

### 4.6 Future work

Several promising avenues for future research emerge from this study. First, integrating multimodal data sources could substantially enhance predictive accuracy. This includes incorporating fluid biomarkers (e.g., cerebrospinal fluid or plasma α-synuclein, tau, and neurofilament light chain), genetic markers (e.g., APOE genotype and GBA mutations), and advanced neuroimaging features (e.g., structural MRI volumetrics, diffusion tensor imaging metrics, and functional connectivity patterns), a strategy validated in numerous high-impact studies (Zhu et al., 2021; Zhu Y. et al., 2024; Hosseinzadeh et al., 2023; Schrag et al., 2017).

Second, investigating the underlying pathophysiological mechanisms linking clinical variables to cognitive decline represents a critical research priority. Such investigations could leverage network connectivity analyses to probe circuit-level dysfunction or examine neuroinflammatory and synaptic dysfunction indicators to elucidate the biological pathways driving PD-MCI development (Baggio et al., 2014; Aarsland et al., 2021; Wang et al., 2025).

Third, addressing the methodological challenges highlighted by our counterintuitive disease duration findings requires analytical approaches specifically designed for time-dependent data and participant attrition. Advanced statistical methods like survival analysis or mixed-effects models for longitudinal data could better account for these complexities and provide more robust insights into disease progression patterns (Liu et al., 2017).

Finally, developing dynamic prediction models represents a paramount future direction. Such models would incorporate longitudinal changes in clinical and biomarker features over time, enabling more accurate, updated risk assessments as patients progress through disease stages. This approach would facilitate the prediction of individual cognitive decline trajectories rather than static point-in-time classification, ultimately providing clinicians with personalized, time-sensitive predictions to guide treatment and counseling (Schrag et al., 2017).

## 5 Conclusion

This study successfully developed and validated machine learning models for the classification of PD-MCI using a comprehensive clinical dataset from the PPMI. Our investigation demonstrates that a parsimonious set of seven readily available clinical features can achieve meaningful discriminative performance for PD-MCI classification, with linear models (SVM and LR) demonstrating superior overall performance with AUC-ROC of 0.7252 and AUC-PR of 0.5008. The consistent identification of age, years of education, and disease duration as the most salient predictors across all models and analytical methodologies confirms established risk factors while providing robust evidence for their clinical utility in screening applications. Notably, the role of certain clinical indicators (such as disease duration) in our models reveals potential data selection biases inherent in longitudinal cohort studies, warranting further investigation.

The methodological rigor of our approach, including subject-level data splitting to prevent data leakage, comprehensive feature selection, and extensive hyperparameter optimization, ensures the reliability and generalizability of our findings. Through multiple validation strategies, including pseudo-external validation by clinical site splitting and cross-validation of feature selection results using diverse methodological approaches (filter, wrapper and embedded methods), we confirmed the robustness of our models and the reliability of our conclusions. The high sensitivity values achieved through threshold optimization (up to 0.8150 for RF under F1-score optimization) demonstrate the potential clinical utility of these models as effective screening tools for early identification of PD-MCI patients. The integration of SHAP analysis enhances model interpretability and clinical trust by providing transparent insights into individual feature contributions to predictions.

While our purely clinical approach offers practical advantages in terms of accessibility and cost-effectiveness compared to multimodal approaches incorporating neuroimaging or biomarker data, future research should focus on integrating these complementary data sources to further enhance predictive accuracy. The development of dynamic prediction models incorporating longitudinal changes and the validation of these models in diverse clinical populations represent important next steps toward implementing these tools in routine clinical practice. Ultimately, this work provides a solid foundation for the development of clinical decision support systems that can facilitate early detection and intervention for PD-MCI, potentially improving patient outcomes through timely therapeutic interventions and care planning.

## Data Availability

Publicly available datasets were analyzed in this study. This data can be found here: Parkinson's Progression Markers Initiative (PPMI) database (https://www.ppmi-info.org).
