# Supplementary material for: Diagnostic classification of mild cognitive impairment in Parkinson's disease using subject-level stratified machine-learning analysis
Source: Front Aging Neurosci. 2025 Oct 22;17:1687925. doi: 10.3389/fnagi.2025.1687925 (PMC12586011; doi:10.3389/fnagi.2025.1687925)
Supplement: Supplementary file 1 [file Data_Sheet_1.pdf]

Supplementary Material

This supplementary material details a series of experiments or results designed to validate the machine learning models used for predicting Parkinson's disease with mild cognitive impairment (PD-MCI).

- **Section 1** outlines the hyperparameter optimization process from the main experiment, defining the comprehensive search spaces for the Logistic Regression (LR), Support Vector Machine (SVM), Random Forest (RF), and XGBoost models, and presents the optimal hyperparameter combinations obtained through Bayesian optimization.

The subsequent supplementary experiments aim to validate the models' robustness and generalizability from various perspectives:

- **Supplementary Experiment I** implements a more stringent site-level split validation strategy to simulate the real-world performance of the models when deployed across different clinical centers.
- **Supplementary Experiment II** evaluates the impact of the feature selection process itself by training the models on all 12 original features without prior selection.
- **Supplementary Experiment III** conducts an ablation study, assessing the performance of a more parsimonious model using only the top 5 predictive features.
- **Supplementary Experiment IV** provides a systematic comparison of multiple feature selection methods—including Filter, Wrapper, and Embedded approaches—to confirm the stability and reliability of the predictors identified in the main experiment.

The complete source code and implementation details for both the main and supplementary experiments have been made publicly available at: `https://github.com/yuzhounh/PD-MCI-Classification`. Running these codes allows for full reproduction of the results reported in this study.

1 MAIN EXPERIMENT: HYPERPARAMETER OPTIMIZATION

Hyperparameter tuning for each model was conducted using Bayesian optimization within a subject-level stratified 10-fold cross-validation scheme on the training set. This validation framework ensures that parameter tuning is robust and does not lead to overfitting on the training data, while maintaining the integrity of subject-level data separation established during the initial data splitting procedure.

We conducted hyperparameter tuning for each model using Bayesian optimization within a subject-level stratified 10-fold cross-validation framework on the training set. The area under the precision-recall curve (AUC-PR) was selected as the optimization objective, which is particularly appropriate for imbalanced datasets as it emphasizes performance on the minority class and provides more informative assessment than traditional accuracy-based metrics. This robust framework ensures that parameter tuning does not lead to overfitting while maintaining the integrity of subject-level data separation.

Table S1 presents the comprehensive hyperparameter search spaces utilized in the Bayesian optimization process for each machine learning algorithm. The search spaces were systematically designed to balance thorough exploration with computational efficiency. For categorical parameters, we included all relevant options supported by each algorithm, while for continuous and integer parameters, we defined ranges based

on established best practices and preliminary experiments. The parameter ranges ensure comprehensive coverage of the optimization landscape while maintaining practical computational constraints, incorporating both commonly used parameter ranges and extended boundaries to capture optimal configurations across diverse scenarios.

Table S1. Hyperparameter Search Spaces for Bayesian Optimization

Model	Hyperparameter	Search Space	Type
LR	penalty	[l1, l2, elasticnet, none]	Categorical
	solver	[liblinear, lbfgs, newton-cg, sag, saga]	Categorical
	C	[10^{-4}, 1]	Log-uniform
	l1_ratio	[0, 1]	Uniform
SVM	kernel	[linear, rbf, poly, sigmoid]	Categorical
	C	[10^{-4}, 1]	Log-uniform
	gamma	[10^{-4}, 1]	Log-uniform
	degree	[2, 5]	Integer
	coef0	[0, 10]	Uniform
RF	n_estimators	[50, 500]	Integer
	max_depth	[3, 20] or none	Integer
	min_samples_split	[2, 20]	Integer
	min_samples_leaf	[1, 30]	Integer
	max_features	[0.1, 1]	Uniform
XGBoost	n_estimators	[50, 500]	Integer
	learning_rate	[0.01, 0.3]	Log-uniform
	max_depth	[2, 6]	Integer
	subsample	[0.6, 1]	Uniform
	colsample_bytree	[0.6, 1]	Uniform
	reg_alpha	[10^{-4}, 1]	Log-uniform
	reg_lambda	[1, 10]	Log-uniform
	gamma	[0, 0.5]	Uniform
	min_child_weight	[1, 20]	Integer
	scale_pos_weight	[1, 5]	Uniform

Note: **Hyperparameter descriptions: LR:** penalty (regularization type), solver (optimization algorithm), C (inverse regularization strength), l1_ratio (elasticnet mixing parameter). **SVM:** kernel (kernel function), C (regularization parameter), gamma (kernel coefficient), degree (polynomial degree), coef0 (independent term in kernel). **RF:** n_estimators (number of trees), max_depth (maximum tree depth), min_samples_split (minimum samples to split node), min_samples_leaf (minimum samples in leaf), max_features (fraction of features per split). **XGBoost:** n_estimators (number of boosting rounds), learning_rate (step size shrinkage), max_depth (maximum tree depth), subsample (fraction of samples per tree), colsample_bytree (fraction of features per tree), reg_alpha (L1 regularization), reg_lambda (L2 regularization), gamma (minimum loss reduction), min_child_weight (minimum sum of instance weight in child), scale_pos_weight (balancing of positive/negative weights).
Valid parameter combinations: LR: l1 penalty requires solver $\in$ {liblinear, saga}; l2 penalty allows solver $\in$ {liblinear, lbfgs, newton-cg, sag, saga}; elasticnet penalty requires solver = saga; none penalty allows solver $\in$ {lbfgs, newton-cg, sag, saga}; l1_ratio is only used when penalty = elasticnet. **SVM:** gamma and degree are only applicable for non-linear kernels (rbf, poly, sigmoid); coef0 is only used for poly and sigmoid kernels; degree is only used for poly kernel. **RF:** max_depth can be integer value [3, 20] or none (unlimited depth); all other parameters are always applicable. **XGBoost:** all parameters are compatible with each other; scale_pos_weight is particularly useful for imbalanced datasets.

Table S2 presents the optimal hyperparameter configurations that yielded the best cross-validation performance for each algorithm. The LR model achieved optimal performance using L2 regularization with the newton-cg solver and a relatively small regularization strength (C = 3.815×10^{-3}). The SVM model performed best with a linear kernel and similarly low regularization parameter (C = 1.199×10^{-3}), indicating that simpler, more regularized models were favored for this dataset. The RF model utilized a moderate number of estimators (374) with controlled tree depth (6) and conservative splitting criteria. The XGBoost model employed a relatively shallow architecture (max_depth = 2) with moderate learning

rate (6.278×10^{-2}) and strong L2 regularization (reg_lambda = 8.896), along with class balancing (scale_pos_weight = 1.852) to handle the imbalanced dataset.

Table S2. Optimal Hyperparameters Obtained via Bayesian Optimization

Algorithm	Hyperparameter	Value
LR	penalty	l2
	solver	newton-cg
	C	3.815×10^{-3}
SVM	kernel	linear
	C	1.199×10^{-3}
RF	n_estimators	374
	max_depth	6
	min_samples_split	18
	min_samples_leaf	28
	max_features	6.009×10^{-1}
XGBoost	n_estimators	280
	learning_rate	6.278×10^{-2}
	max_depth	2
	subsample	9.904×10^{-1}
	colsample_bytree	6.151×10^{-1}
	reg_alpha	4.610×10^{-2}
	reg_lambda	8.896
	gamma	4.254×10^{-2}
	min_child_weight	18
	scale_pos_weight	1.852

2 SUPPLEMENTARY EXPERIMENT I: SITE-LEVEL SPLIT VALIDATION

2.1 Introduction and Motivation

2.1.1 Parkinson's Disease Dataset Landscape

In clinical machine learning research, evaluating model performance on external datasets from diverse geographical regions and healthcare systems is crucial for establishing generalizability and robustness in real-world deployment scenarios. However, obtaining access to well-matched, high-quality, and publicly available datasets for rigorous external validation remains a substantial methodological challenge.

While other high-quality Parkinson's Disease (PD) research cohorts exist, such as the Parkinson's Disease Biomarkers Program (PDBP), the Accelerating Medicines Partnership - Parkinson's Disease (AMP-PD), and Tracking Parkinson's (UK), we were unable to secure data access permissions for them. In contrast, the Parkinson's Progression Markers Initiative (PPMI) dataset is significantly more accessible to the academic research community, with a well-established and relatively streamlined application process that is more frequently approved. This is largely due to the PPMI being a large, multi-center study with a large number of participants and a well-established data collection and sharing protocol.

As evidenced by publication counts from Google Scholar (Table S3), the PPMI dataset is far more widely used and cited within the scientific community than other comparable cohorts. This has established PPMI as a de facto benchmark dataset for developing and validating new methods in PD research. Basing our study on PPMI ensures that our results are comparable, reproducible, and immediately relevant to the vast majority of researchers in this field.

Table S3. Comparison of Parkinson's Disease (PD) Datasets

Abbreviation	Full Name	Link	Count
PPMI	Parkinson's Progression Markers Initiative	`https://www.ppmi-info.org/`	19,800
PDBP	Parkinson's Disease Biomarkers Program	`https://pdbp.ninds.nih.gov/`	2,370
AMP-PD	Accelerating Medicines Partnership - PD	`https://amp-pd.org/`	769
TP-UK	Tracking Parkinson's (UK)	`https://www.parkinsons.org.uk`	576

2.1.2 Site-level Split Validation Strategy

Due to the inability to directly obtain external Parkinson's disease datasets for true external validation, we implemented a more stringent, site-level stratified sample splitting approach to create a "pseudo-external" validation set for model generalization assessment. This methodology ensures that the training and testing sets originate from completely different clinical sites, thereby simulating real-world application scenarios where models are deployed across different healthcare institutions.

To evaluate the model's generalization performance when deployed to new, previously unseen clinical environments, we designed this clinical center-based splitting strategy to simulate external validation. This experiment is conducted on the same dataset as the main paper, with the same machine learning models and evaluation metrics, providing a rigorous assessment of cross-site generalizability.

Given the difficulty of obtaining a true external dataset, we devised this more stringent splitting strategy within the PPMI cohort to simulate external validation. By implementing a site-level splitting strategy, we created a "pseudo-external" test set to rigorously assess our model's generalization capabilities across different clinical environments.

2.2 Materials and Methods

2.2.1 Data Distribution Across Sites

The core methodology of this supplementary experiment leverages the multi-center nature of the PPMI study. Data for the PPMI cohort were collected from numerous clinical sites worldwide, which may have subtle variations in equipment, protocols, or patient demographics. Specifically, the dataset used in this study comprises data from 51 distinct sites, with the distribution of subjects and samples detailed in Table S4.

Importantly, our analysis confirmed that each subject in the PPMI cohort is exclusively associated with a single clinical site—no subject visited multiple sites during the study period. This one-to-one mapping between subjects and sites ensures that our site-level splitting strategy creates truly independent training and test sets without any subject overlap between sites.

2.2.2 Site-level Stratified Splitting

To simulate external validation, we employed a site-level stratified splitting approach where data from a subset of sites were used for training and data from the remaining, unseen sites for testing. This methodology effectively simulates the model's performance when deployed in entirely new clinical environments, providing a rigorous assessment of generalization capability across different healthcare settings.

The key distinction between this supplementary experiment and the main study lies in the data splitting methodology. While the main paper utilized a subject-level stratified random split, where all longitudinal

Table S4. Distribution of Subjects and Samples Across PPMI Sites

Site	No. Subjects	No. Samples	Site	No. Subjects	No. Samples	Site	No. Subjects	No. Samples
10	12	39	27	7	22	60	2	5
11	2	5	28	34	139	61	29	117
12	3	7	29	12	43	62	33	134
13	32	132	30	2	6	63	4	10
14	33	136	31	43	182	64	9	31
15	3	9	32	14	50	65	4	11
16	2	5	33	36	145	66	20	79
17	5	15	34	18	69	67	33	136
18	4	12	35	18	69	68	2	4
19	42	183	36	8	28	69	20	78
20	17	58	37	16	58	70	9	29
21	17	62	38	2	6	71	22	90
22	24	96	39	11	37	72	12	44
23	6	19	40	4	11	73	2	4
24	20	73	41	5	15	75	4	12
25	2	4	42	17	63	76	3	9
26	129	556	44	2	6	79	1	1
**Total**	**896**	**3154**						

visits from each individual subject were allocated exclusively to either the training or test set, this experiment implements a site-level stratified split. In the site-level approach, all subjects recruited from each clinical site, along with their complete visit histories, are assigned entirely to either the training or test partition.

This site-level stratification strategy was consistently applied across two critical stages of the analysis: (1) the primary division for creating training and test sets (70:30 ratio) used in final model evaluation, and (2) the 10-fold cross-validation procedure conducted within the training set for both feature selection and hyperparameter optimization.

The complete source code for this experiment is available at: `https://github.com/yuzhounh/PD-MCI-Classification/tree/main/supplementary_experiment_1`.

2.3 Results and Analysis

2.3.1 Feature Selection

Using the site-level split training set, LASSO logistic regression was applied for feature selection. As shown in Figure S1, an optimal regularization parameter of $\lambda = 19.9526$ was identified. The feature weights of the final LASSO model are displayed in Figure S2. At this strength, LASSO selected a parsimonious subset of 8 predictive features from the initial 12 candidates: Age, EDUCYRS, Duration, Sex, UPDRS-I, GDS, UPDRS-IV, and UPDRS-III.

Compared to the results in Figure 4 of the main paper, the feature importance hierarchy showed remarkable consistency: Age, EDUCYRS, and Duration maintained their positions as the three most important predictors with identical ranking order. Sex, UPDRS-I, and GDS formed the second tier of importance with slight variations in their relative ordering. UPDRS-III and UPDRS-IV were retained as the least important features, with UPDRS-IV being an additional feature selected in this site-level analysis that was not present in the main paper's subject-level split results. This suggests that when accounting for inter-site variability, UPDRS-IV may provide additional, albeit minor, discriminative information.

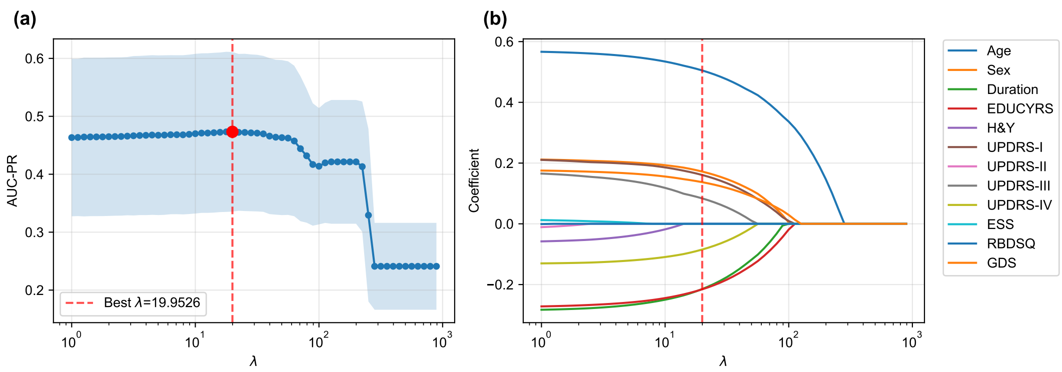

Figure S1. LASSO Logistic Regression Feature Selection based on Site-Level Split. (a) Mean AUC-PR from 10-fold cross-validation across a range of λ values. (b) Coefficient paths for each feature as a function of λ.

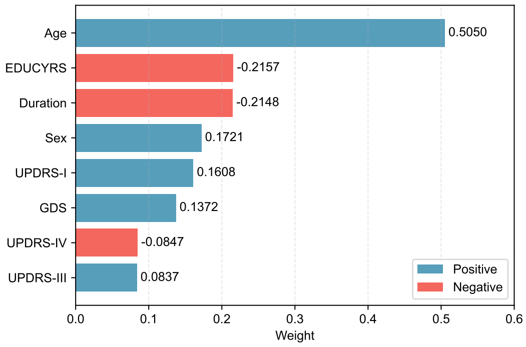

Figure S2. Feature weights for the 8 selected features in the final LASSO model.

2.3.2 Model Performance Evaluation

The four machine learning models were evaluated on the ”pseudo-external” test set using the site-level split approach. Figure S3 presents the ROC and precision-recall curves, demonstrating each model’s discriminative performance. The comprehensive performance evaluation is detailed in Table S5, which provides both threshold-independent metrics (AUC-ROC and AUC-PR) and threshold-dependent metrics under three different threshold selection strategies: the default threshold of 0.5, the F1-score optimized threshold, and the Youden Index optimized threshold.

The results reveal distinct performance patterns across the four models. For threshold-independent metrics, LR achieved the highest performance with an AUC-ROC of 0.7092 and AUC-PR of 0.5134, followed by SVM (AUC-ROC: 0.6898, AUC-PR: 0.4734). The ensemble methods, RF and XGBoost, demonstrated notably lower discriminative capabilities.

Threshold-dependent performance varied significantly across different optimization strategies. Under the default threshold of 0.5, SVM achieved the highest accuracy (0.7210) and precision (0.6327) but suffered from extremely low sensitivity (0.1003), indicating poor detection of PD-MCI cases. In contrast, LR demonstrated more balanced performance with the highest F1-score (0.5149) and Cohen’s Kappa (0.2666), suggesting better overall classification quality.

When thresholds were optimized for F1-score and Youden Index, the performance gaps between LR and SVM narrowed considerably. LR maintained consistent superiority in F1-score and Cohen’s Kappa across

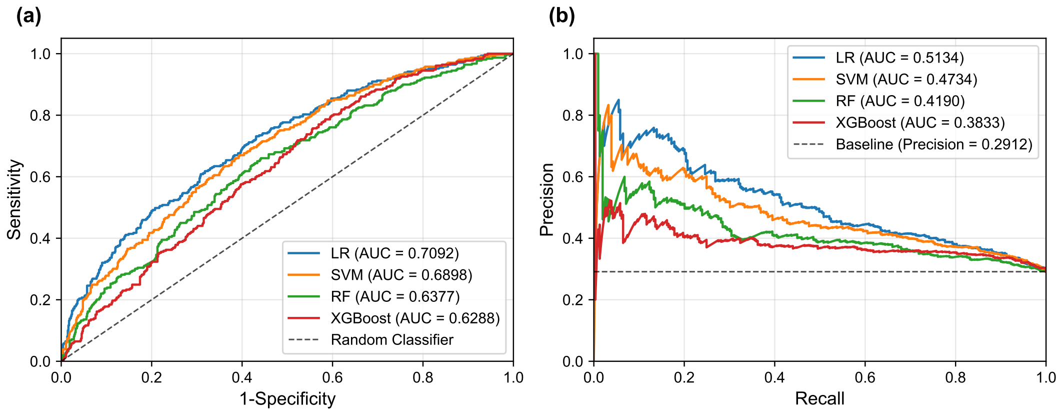

Figure S3. Performance curves of the four machine learning models on the "pseudo-external" test set. (a) ROC curves illustrate the ability to discriminate between PD-MCI and PD-NC. (b) PR curves focus on performance for the minority class (PD-MCI) in an imbalanced dataset.

Table S5. Performance Comparison on Test Data Across Different Threshold Strategies (Site-Level Stratified Splitting).

Threshold	Metric	LR	SVM	RF	XGBoost
	AUC-ROC	**0.7092**	0.6898	0.6377	0.6288
	AUC-PR	**0.5134**	0.4734	0.4190	0.3833
**Default (0.5)**	Accuracy	0.6626	**0.7210**	0.6522	0.5504
	Balanced Accuracy	**0.6485**	0.5382	0.5897	0.5913
	Precision	0.4429	**0.6327**	0.4096	0.3586
	Sensitivity	0.6149	0.1003	0.4401	**0.6893**
	Specificity	0.6822	**0.9761**	0.7394	0.4934
	F1-score	**0.5149**	0.1732	0.4243	0.4718
	Cohen's Kappa	**0.2666**	0.1016	0.1756	0.1436
**F1-Score**	Optimal Threshold	0.4844	0.2249	0.4833	0.6081
	Accuracy	**0.6447**	0.6287	0.6249	0.6258
	Balanced Accuracy	**0.6416**	0.6351	0.5905	0.5673
	Precision	**0.4261**	0.4127	0.3896	0.3750
	Sensitivity	0.6343	**0.6505**	0.5081	0.4272
	Specificity	0.6489	0.6197	0.6729	**0.7074**
	F1-score	**0.5098**	0.5050	0.4410	0.3994
	Cohen's Kappa	**0.2476**	0.2310	0.1661	0.1293
**Youden Index**	Optimal Threshold	0.4900	0.2249	0.4954	0.5720
	Accuracy	**0.6541**	0.6287	0.6437	0.5994
	Balanced Accuracy	**0.6483**	0.6351	0.5866	0.5782
	Precision	**0.4356**	0.4127	0.4006	0.3688
	Sensitivity	0.6343	**0.6505**	0.4498	0.5275
	Specificity	0.6622	0.6197	**0.7234**	0.6290
	F1-score	**0.5165**	0.5050	0.4238	0.4341
	Cohen's Kappa	**0.2614**	0.2310	0.1672	0.1389

all threshold strategies, while SVM showed improved sensitivity at optimized thresholds. This pattern underscores the importance of appropriate threshold selection, particularly for imbalanced datasets where minority class detection is crucial.

Compared to the subject-level split results reported in Figure 5 of the main paper, the site-level split presented a more stringent validation scenario. Most AUC values decreased under this challenging evaluation approach, reflecting the difficulty of generalizing across different clinical sites. However, a notable

exception emerged: the LR model's AUC-PR improved from 0.4923 to 0.5134, suggesting enhanced capability in identifying PD-MCI cases when accounting for inter-site variability. This improvement in the most clinically relevant metric (AUC-PR for the minority class) provides encouraging evidence for the model's potential real-world applicability.

2.3.3 Feature Importance Analysis

Despite the drop in performance, the overall pattern of feature importance remained highly consistent with the findings of the main paper, as shown in Figures S4 and S5. Across all models and various importance metrics (e.g., coefficients, SHAP values, permutation importance), Age, EDUCYRS, and Duration generally ranked as the most salient predictors of PD-MCI. This consistency underscores the robustness of these key features across different clinical contexts.

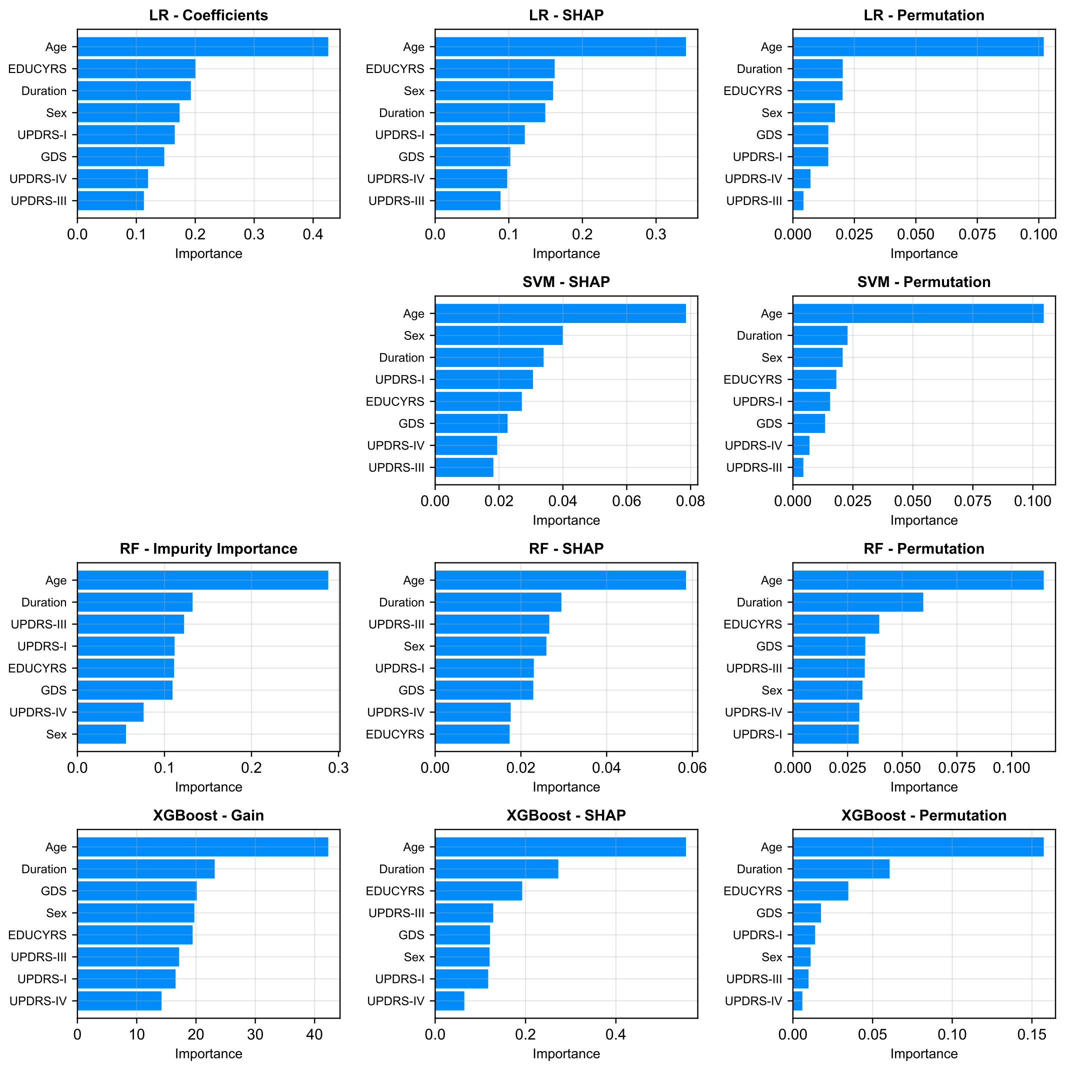

Figure S4. Feature importance comparison across four machine learning models using three evaluation metrics. The SVM model does not display coefficient weights as Bayesian hyperparameter optimization selected the RBF kernel, which does not provide directly interpretable linear coefficients like linear SVM.

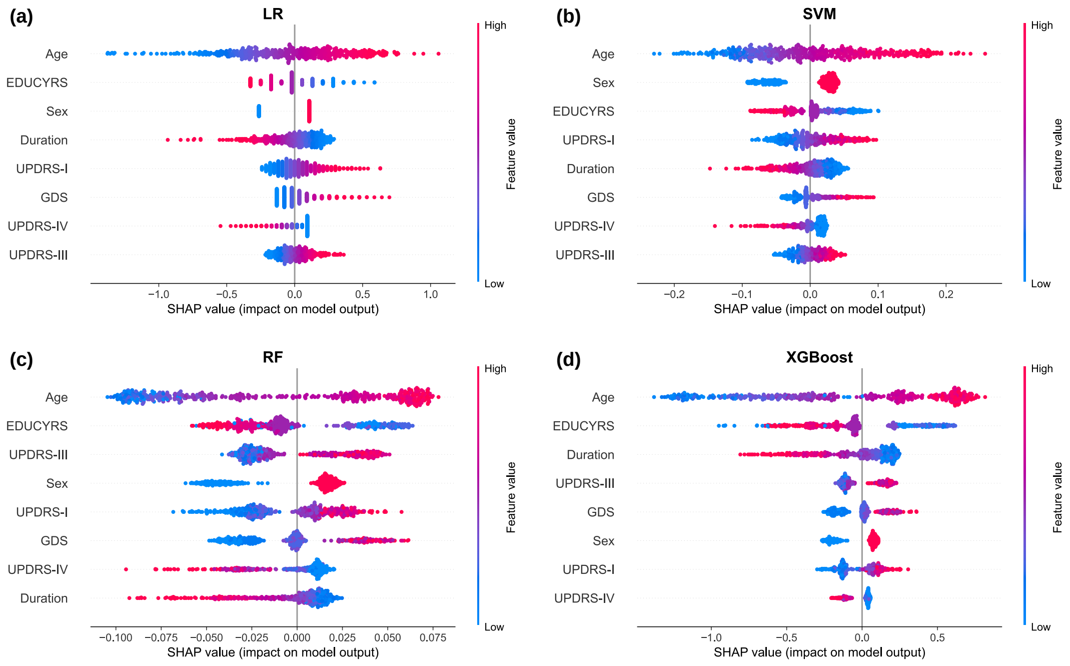

Figure S5. SHAP summary plots for the four models, visualizing the impact (magnitude and direction) of each feature on model output.

2.4 Comparison with Main Paper

In terms of feature selection, the site-split LASSO model identified 8 predictive features, representing one additional feature compared to the 7 features selected by the subject-split model in the main paper. The additional feature was ”UPDRS-IV” (Motor Complications), suggesting that when accounting for inter-site variability, motor complications may provide additional, albeit minor, discriminative information for distinguishing between PD-MCI and PD-NC patients.

Regarding model performance, the site-level validation approach presented a more challenging evaluation scenario as anticipated. While both AUC-ROC and AUC-PR values generally decreased across the four models compared to the subject-level split results in the main paper, a notable exception emerged in the most clinically relevant metric. The highest AUC-PR value achieved in this experiment (0.5134, obtained by LR) demonstrated a meaningful improvement over the best performance reported in the main paper (0.5008, achieved by SVM). This improvement in AUC-PR is particularly significant given that this metric specifically evaluates performance on the minority class (PD-MCI) in our imbalanced dataset, making it the most clinically relevant indicator of diagnostic utility.

The observed performance patterns reflect the inherent challenges of the site-level validation approach. By ensuring that all subjects from each clinical site are allocated exclusively to either training or test sets, this methodology simulates the realistic scenario of deploying models across different clinical centers with potentially distinct patient populations and data collection protocols. While most metrics showed expected degradation, the improvement in the key AUC-PR metric suggests that the site-level approach may actually enhance the models' ability to identify PD-MCI cases, providing a more conservative yet clinically meaningful estimate of real-world generalization capabilities.

Most importantly, despite the mixed performance changes, the feature importance analysis revealed remarkable consistency with the main paper findings. The most important predictors (Age, EDUCYRS, and

Duration) remained unchanged across both validation approaches. This finding significantly strengthens our confidence in the study's conclusions, indicating that these core predictors are not artifacts of the specific data distribution within the PPMI cohort but represent robust patterns that hold across diverse patient subpopulations and clinical contexts.

2.5 Conclusion

Through this site-split experiment simulating external validation, we assessed our models' generalization capabilities under a more challenging scenario. The results demonstrate that while model performance experiences a modest decline, it retains meaningful discriminative power. Most importantly, the core predictive features remain highly stable. This provides stronger evidence supporting the potential for these models to be generalized and applied in real-world, multi-center clinical settings.

3 SUPPLEMENTARY EXPERIMENT II: FEATURE RANKING BY FOUR MODELS

3.1 Motivation

The main experiment employed LASSO feature selection to identify the most predictive features before model training. To validate this methodological choice and assess whether feature selection introduces bias or overlooks important predictors, we conducted this supplementary experiment using all 12 original features without prior LASSO selection. This comparison allows us to evaluate: (1) the consistency of feature importance rankings across different approaches, and (2) the performance trade-offs between feature selection and using the complete feature set.

3.2 Methodology

We applied the four models (LR, SVM, RF, XGBoost) directly to all 12 original features, bypassing the LASSO feature selection step. All other experimental protocols remained identical to the main paper: 10-fold stratified cross-validation on the training set, Bayesian optimization targeting AUC-PR maximization, followed by final model training on the complete training set using optimal hyperparameters.

The complete source code for this experiment is available at: `https://github.com/yuzhounh/PD-MCI-Classification/tree/main/supplementary_experiment_2`.

3.3 Results

3.3.1 Feature Importance Analysis

Figure S6 presents the feature importance results across all four models. Remarkably, during Bayesian optimization, the LR algorithm automatically selected ElasticNet regularization as the optimal penalty, which inherently performs feature selection by shrinking coefficients to zero. This resulted in the automatic selection of 6 features with non-zero coefficients, which corresponded to the top 6 features from the main experiment (i.e., all features except the one with the lowest importance ranking). For SVM, Bayesian optimization selected a linear kernel, enabling direct visualization of feature coefficients.

The most striking finding is the remarkable consistency in feature ranking across all models and methodological approaches. Age, EDUCYRS, and Duration consistently emerged as the top three most important predictors, maintaining their dominant positions regardless of whether LASSO feature selection was applied or not. This consistency validates the robustness of these core predictive features and demonstrates that their importance is not an artifact of the specific feature selection methodology employed.

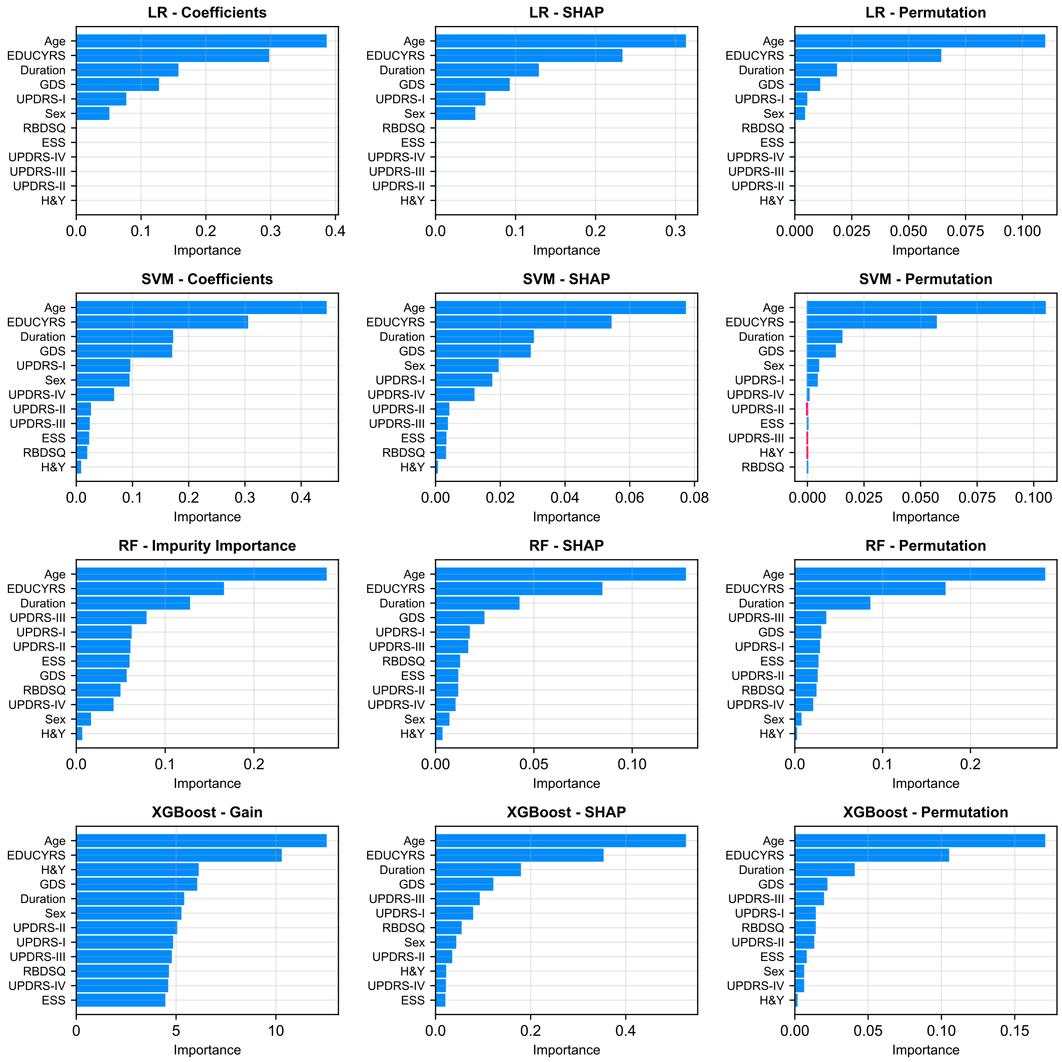

Figure S6. Feature importance comparison across four machine learning models (rows) without prior LASSO feature selection, using all 12 original features. The analysis employs three different evaluation metrics (columns): model-specific importance (Coefficients for LR/SVM, Gini Impurity for RF, and Gain for XGBoost), mean absolute SHAP values, and permutation importance.

3.3.2 Model Performance

Figure S7 and Table S6 presents the comprehensive performance results for all four models trained on the complete set of 12 original features. The ROC curves demonstrate that SVM achieved the best discriminative ability with the highest AUC-ROC (0.7287), followed closely by LR (0.7169). The PR curves reveal that SVM also attained the highest AUC-PR (0.5066), indicating superior performance in identifying the minority class (PD-MCI) in our imbalanced dataset.

Notably, the LR, SVM, and RF models achieved identical optimal thresholds when using both F1-score and Youden Index optimization methods, suggesting convergence to similar decision boundaries. Under the default threshold (0.5), SVM maintained its characteristic pattern of high precision (0.6316) and specificity (0.9675) but extremely low sensitivity (0.1417), as clearly illustrated by its steep PR curve. When thresholds were optimized for F1-score and Youden Index, SVM achieved the best overall performance across most

metrics, including F1-score (0.5408) and Cohen's Kappa (0.2715), demonstrating improved balance between precision and recall.

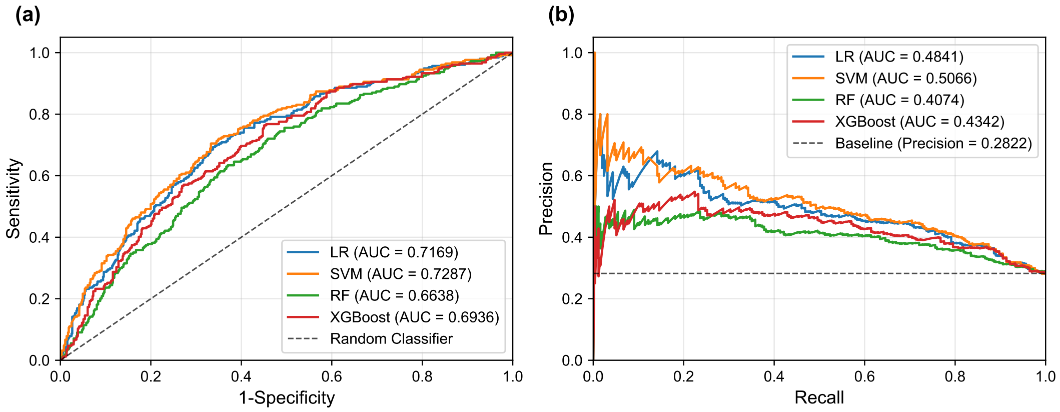

Figure S7. ROC and PR curves for four machine learning models evaluated on the test set. This experiment utilized the complete feature set of all 12 original features without prior LASSO feature selection.

Table S6. Performance Comparison on Test Data Across Different Threshold Strategies (Full Model with 12 Features).

Threshold	Metric	LR	SVM	RF	XGBoost
	AUC-ROC	0.7169	**0.7287**	0.6638	0.6936
	AUC-PR	0.4841	**0.5066**	0.4074	0.4342
**Default (0.5)**	Accuracy	0.6633	**0.7344**	0.6600	0.7244
	Balanced Accuracy	**0.6747**	0.5546	0.6115	0.5620
	Precision	0.4395	**0.6316**	0.4150	0.5333
	Sensitivity	**0.7008**	0.1417	0.5000	0.1890
	Specificity	0.6486	**0.9675**	0.7229	0.9350
	F1-score	**0.5402**	0.2315	0.4536	0.2791
	Cohen's Kappa	**0.2960**	0.1428	0.2099	0.1542
**F1-Score**	Optimal Threshold	0.4766	0.2332	0.4100	0.2778
	Accuracy	**0.6256**	0.6244	0.5922	0.5778
	Balanced Accuracy	0.6699	**0.6727**	0.6287	0.6378
	Precision	0.4126	**0.4129**	0.3811	0.3788
	Sensitivity	0.7717	**0.7835**	0.7126	0.7756
	Specificity	**0.5681**	0.5619	0.5449	0.5000
	F1-score	0.5377	**0.5408**	0.4966	0.5090
	Cohen's Kappa	0.2688	**0.2715**	0.2037	0.2091
**Youden Index**	Optimal Threshold	0.4766	0.2332	0.4100	0.2702
	Accuracy	**0.6256**	0.6244	0.5922	0.5700
	Balanced Accuracy	0.6699	**0.6727**	0.6287	0.6383
	Precision	0.4126	**0.4129**	0.3811	0.3762
	Sensitivity	0.7717	**0.7835**	0.7126	0.7953
	Specificity	**0.5681**	0.5619	0.5449	0.4814
	F1-score	0.5377	**0.5408**	0.4966	0.5107
	Cohen's Kappa	0.2688	**0.2715**	0.2037	0.2068

3.3.3 Comparison with Main Paper

When comparing with the main paper results (which used LASSO feature selection), several important patterns emerge. Using all 12 features led to modest improvements in some metrics: SVM's AUC-PR

increased from 0.5008 to 0.5066 and its F1-score improved from 0.5303 to 0.5408. However, these improvements came at the cost of increased model complexity, requiring all 12 features instead of the 7 selected by LASSO.

The most significant finding is the remarkable stability of feature rankings across both approaches. Age, EDUCYRS, and Duration consistently maintained their positions as the top three predictors, demonstrating that LASSO successfully identified the most informative features rather than introducing selection bias. This consistency provides strong validation for the feature selection methodology and strengthens confidence in the clinical relevance of these predictors.

Furthermore, the characteristic performance patterns of each algorithm remained consistent between the two approaches. SVM continued to exhibit high precision but low sensitivity under default thresholds, while LR maintained more balanced performance across different metrics. This stability in model behavior patterns validates the robustness of our findings and suggests that the identified relationships between features and outcomes are genuine rather than artifacts of the feature selection process.

To statistically validate the performance comparison between the 7-feature model (with LASSO feature selection) and the 12-feature model (without LASSO feature selection), we conducted DeLong tests to assess the significance of AUC-ROC differences. Table S7 presents the comprehensive statistical comparison results.

Table S7. DeLong Test Results Comparing 7-Feature Model (Model A) vs. 12-Feature Model (Model B) Performance.

Algorithm	Model A AUC	Model B AUC	Difference	95% CI	Z Statistic	p-value
LR	0.7241	0.7169	0.0072	[0.0033, 0.0112]	3.5659	0.0004
SVM	0.7252	0.7287	-0.0035	[-0.0087, 0.0018]	-1.3045	0.1921
RF	0.6882	0.6638	0.0244	[0.0079, 0.0409]	2.8967	0.0038
XGBoost	0.6967	0.6936	0.0031	[-0.0097, 0.0158]	0.4715	0.6373

The DeLong test results reveal distinct algorithm-specific patterns in the comparison between the 7-feature LASSO-selected model and the full 12-feature model. For LR and RF, the 7-feature model demonstrates statistically significant superiority ($p < 0.01$), with AUC improvements of 0.72% and 2.44% respectively. This finding suggests that LASSO feature selection not only reduces model complexity but actually enhances predictive performance by eliminating noise from less informative features. In contrast, SVM and XGBoost show no statistically significant differences between the two models ($p > 0.05$), indicating these algorithms maintain robust performance regardless of feature dimensionality. Notably, SVM exhibits a slight performance decrease with the reduced feature set (AUC difference: -0.0035), though this difference lacks statistical significance.

3.4 Discussion

This experiment provides compelling validation of our LASSO-based feature selection methodology. The convergence of LR with ElasticNet regularization to the same 6-feature subset identified by LASSO demonstrates that our feature selection captured the most informative predictors rather than introducing methodological bias.

The performance comparison reveals that while using all 12 features provides modest improvements, the gains are relatively small compared to the increased model complexity. Given that our dataset contains only 12 features, the benefits of feature selection are less pronounced than they would be in high-dimensional

datasets. However, LASSO successfully simplified the model from 12 to 7 features while maintaining comparable performance, which is valuable for clinical applications as it reduces data collection burden and enhances interpretability.

The consistency of feature importance rankings across different methodological approaches strengthens confidence in our findings. The stability of Age, EDUCYRS, and Duration as the most important predictors, regardless of feature selection strategy, provides robust evidence for their clinical relevance in PD-MCI prediction.

Furthermore, this experiment demonstrates why LASSO was preferred over other feature selection approaches. While models like SVM, RF, and XGBoost can rank feature importance, they require subjective decisions about the number of features to retain. The automatic feature selection capability of L1-regularized methods eliminates this subjectivity while maintaining robust predictive performance.

3.5 Conclusion

This supplementary experiment validates our methodological choices and strengthens confidence in the generalizability of our findings. The identified predictive features demonstrate remarkable stability across different approaches, and LASSO feature selection successfully balanced model simplicity with predictive performance, making it well-suited for clinical applications.

4 SUPPLEMENTARY EXPERIMENT III: THE PARSIMONIOUS 5-FEATURE MODEL

4.1 Motivation

Given the strong consensus across all feature selection methodologies identifying Age, EDUCYRS, and Duration as the most critical predictors, we conducted an ablation study to evaluate whether a more parsimonious model using only the top-ranked features could maintain comparable performance to the full 7-feature model.

4.2 Methodology

From the seven consensus features identified in the main experiment, we systematically removed the two lowest-importance features (UPDRS-III and sex), resulting in a reduced feature set of five features: Age, EDUCYRS, Duration, GDS, and UPDRS-I. The same machine learning pipeline and evaluation framework used in the main experiment were applied.

The complete source code for this experiment is available at: `https://github.com/yuzhounh/PD-MCI-Classification/tree/main/supplementary_experiment_3`.

4.3 Results

Figure S8 presents the ROC and PR curves for all four machine learning models using the reduced 5-feature set. LR achieves the highest AUC-ROC of 0.7108, followed by XGBoost (0.6973), SVM (0.6948), and RF (0.6854). For AUC-PR, LR also achieves the highest value of 0.4730, followed by RF (0.4548), XGBoost (0.4322), and SVM (0.4371).

Table S8 provides comprehensive performance evaluation across different threshold optimization strategies. Notably, the F1-score optimization and Youden Index optimization yield remarkably similar results across all models, with LR and RF achieving identical performance metrics under both criteria. This convergence between the two optimization approaches indicates robust threshold stability. Furthermore,

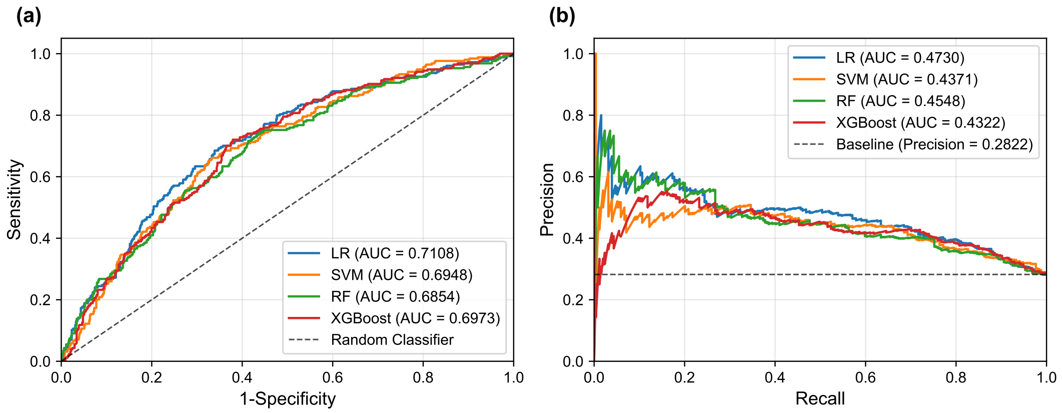

Figure S8. ROC and PR curves for four machine learning models evaluated on the test set using the parsimonious 5-feature model. The curves demonstrate the classification performance of each algorithm when trained with the reduced feature set consisting of Age, EDUCYRS, Duration, GDS, and UPDRS-I.

LR consistently demonstrates superior performance under both threshold optimization strategies, achieving the highest F1-score (0.5251) and Cohen's kappa (0.2594), indicating the most balanced classification performance across different optimization criteria.

Table S8. Performance Comparison on Test Data Across Different Threshold Strategies (Parsimonious Model with 5 Features).

Threshold	Metric	LR	SVM	RF	XGBoost
	AUC-ROC	**0.7108**	0.6948	0.6854	0.6973
	AUC-PR	**0.4730**	0.4371	0.4548	0.4322
**Default (0.5)**	Accuracy	0.6622	**0.7133**	0.6267	0.6489
	Balanced Accuracy	**0.6703**	0.5232	0.6396	0.6682
	Precision	0.4375	**0.4583**	0.4028	0.4269
	Sensitivity	0.6890	0.0866	0.6693	**0.7126**
	Specificity	0.6517	**0.9598**	0.6099	0.6238
	F1-score	**0.5352**	0.1457	0.5030	0.5339
	Cohen's Kappa	**0.2901**	0.0615	0.2325	0.2796
**F1-Score**	Optimal Threshold	0.4852	0.2284	0.4576	0.4561
	Accuracy	**0.6322**	0.6178	0.5478	0.5967
	Balanced Accuracy	**0.6590**	0.6465	0.6193	0.6486
	Precision	**0.4131**	0.4004	0.3612	0.3908
	Sensitivity	0.7205	0.7126	**0.7835**	0.7677
	Specificity	**0.5975**	0.5805	0.4551	0.5294
	F1-score	**0.5251**	0.5127	0.4944	0.5179
	Cohen's Kappa	**0.2594**	0.2370	0.1761	0.2299
**Youden Index**	Optimal Threshold	0.4852	0.2244	0.4576	0.4451
	Accuracy	**0.6322**	0.6100	0.5478	0.5900
	Balanced Accuracy	**0.6590**	0.6483	0.6193	0.6499
	Precision	**0.4131**	0.3970	0.3612	0.3883
	Sensitivity	0.7205	0.7362	0.7835	**0.7874**
	Specificity	**0.5975**	0.5604	0.4551	0.5124
	F1-score	**0.5251**	0.5159	0.4944	0.5202
	Cohen's Kappa	**0.2594**	0.2355	0.1761	0.2285

To statistically validate the performance comparison between the full 7-feature model and the parsimonious 5-feature model, we conducted DeLong tests to assess the significance of AUC-ROC differences. Table S9 presents the comprehensive statistical comparison results.

Table S9. DeLong Test Results Comparing 7-Feature Model (Model A) vs. 5-Feature Model (Model B) Performance.

Algorithm	Model A AUC	Model B AUC	Difference	95% CI	Z Statistic	p-value
LR	0.7241	0.7108	0.0133	[0.0066, 0.0199]	3.8960	0.0001
SVM	0.7252	0.6948	0.0304	[0.0089, 0.0519]	2.7679	0.0056
RF	0.6882	0.6854	0.0027	[-0.0090, 0.0145]	0.4582	0.6468
XGBoost	0.6967	0.6973	-0.0006	[-0.0134, 0.0123]	-0.0856	0.9318

The DeLong test results reveal algorithm-specific patterns in performance degradation. For LR and SVM, the 7-feature model demonstrates statistically significant superiority over the 5-feature model ($p < 0.05$), with AUC reductions of 1.8% and 4.2% respectively. However, the absolute differences remain clinically modest, with 95% confidence intervals indicating relatively small effect sizes. In contrast, RF and XGBoost show no statistically significant differences between the two models ($p > 0.05$), suggesting these ensemble methods maintain robust performance even with reduced feature dimensionality. Notably, XGBoost exhibits virtually identical performance between both models (AUC difference: -0.0006), indicating exceptional stability to feature reduction.

4.4 Conclusion

This ablation study reveals important insights about the trade-off between model parsimony and predictive performance. When reducing from the original 7 features to 5 features, the DeLong test results demonstrate algorithm-specific responses: LR and SVM show statistically significant performance degradation ($p < 0.01$), while RF and XGBoost maintain robust performance with no significant differences ($p > 0.05$). Notably, the top 2 AUC values across all algorithms were achieved with the full 7-feature model. Although UPDRS-III and sex exhibit relatively lower importance rankings, their removal negatively impacts overall model performance, particularly for linear algorithms. Therefore, while the 5-feature model offers reduced complexity, retaining all 7 features represents the optimal choice for maximizing predictive performance. This finding suggests that even seemingly less important features contribute meaningful information to the classification task, and the pursuit of optimal clinical prediction accuracy justifies the inclusion of the complete feature set.

5 SUPPLEMENTARY EXPERIMENT IV: COMPARISON OF FEATURE SELECTION

5.1 Introduction and Motivation

Feature selection is critical for building robust and interpretable machine learning models, especially in clinical applications where transparency and reliability are essential. To validate our LASSO-based feature selection approach and assess the stability of identified predictors, we conducted a comprehensive comparative evaluation across all three major feature selection categories: Filter, Wrapper, and Embedded methods.

We employed four statistical tests for filter methods: ANOVA F-test, Chi-square test, Pearson correlation, and Mutual Information. For wrapper and embedded methods, we used four machine learning algorithms:

LR, linear SVM, RF, and XGBoost, all with default parameters to avoid computational complexity from simultaneous hyperparameter tuning.

Different validation strategies were applied based on computational requirements and methodological characteristics. Filter methods, being model-independent and computationally efficient, were evaluated on the entire training dataset to capture comprehensive statistical relationships. Wrapper and embedded methods, which are computationally intensive, were evaluated using 10-fold cross-validation with subject-level stratified sampling within the training dataset to ensure robust feature selection stability while managing computational complexity.

This validation strategy design reflects each method's inherent characteristics: filter methods provide stable statistical measures without requiring cross-validation, while wrapper and embedded methods benefit from cross-validation to evaluate consistency across data partitions and mitigate model-dependent overfitting risks.

5.2 Filter Methods

Filter methods are performed as a pre-processing step, independent of any machine learning model. They analyze the intrinsic statistical properties of the features and their relationship with the target variable to ”filter out” irrelevant features.

We evaluated four common statistical tests to score and rank each feature. The ANOVA F-test measures the difference in means of a continuous feature across the classes of the target variable. The Chi-Squared Test measures the dependence between categorical features and the target variable. Pearson Correlation measures the linear relationship between a continuous feature and the target variable. Finally, Mutual Information measures the dependency between a feature and the target variable, capturing both linear and non-linear relationships.

Filter methods offer several advantages: they are computationally very fast and model-agnostic, meaning the selected features can be used with any learning algorithm. However, they also have notable disadvantages. They are typically univariate, evaluating each feature independently. This may lead them to overlook features that are not useful on their own but are highly predictive when combined with others.

The results from the four filter methods are presented in Figure S9. A remarkable consistency is observed across all four distinct statistical approaches. Age emerges as the most dominant predictor, achieving the highest scores across all methods. EDUCYRS consistently ranks as the second most important feature, demonstrating strong predictive power across different statistical measures.

Notably, the filter method rankings show excellent concordance with the LASSO feature selection results from the main experiment. Both approaches identify Age and EDUCYRS as the top two predictors, with Age receiving the highest importance and EDUCYRS the second highest. GDS also demonstrates consistent importance across filter methods and ranks fourth in the LASSO selection. Duration and UPDRS-I, which rank third and fifth respectively in the LASSO results, also show substantial scores in the filter analysis, further validating their predictive relevance.

This cross-methodological validation between model-independent filter methods and the LASSO approach provides compelling evidence for the fundamental predictive power of these demographic and clinical variables, strengthening confidence in their clinical significance for PD-MCI classification.

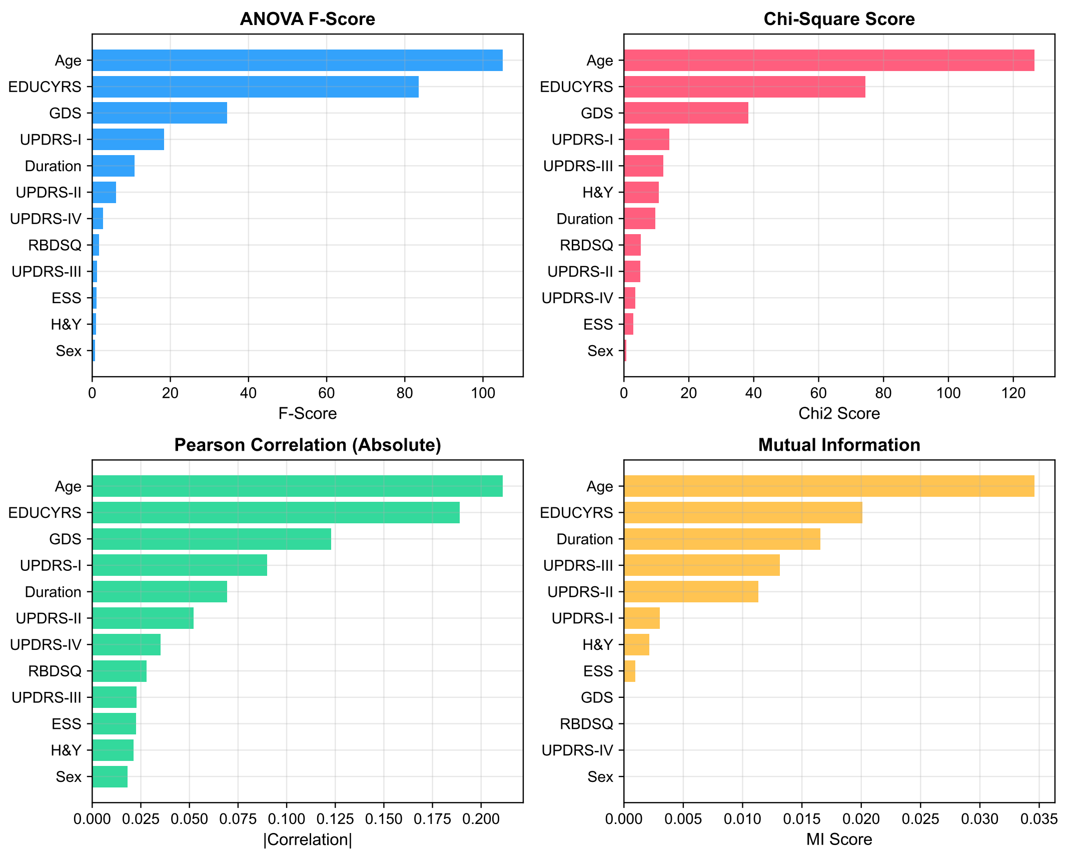

Figure S9. Feature importance ranking according to four different filter methods: ANOVA F-Score, Chi-Square Score, Pearson Correlation (Absolute), and Mutual Information. Higher values indicate greater feature importance. These results were obtained by training on the entire training dataset to capture comprehensive statistical relationships between features and the target variable.

5.3 Wrapper Methods

Wrapper methods ”wrap” the feature selection process around a machine learning model. They treat feature selection as a search problem, where different feature subsets are evaluated based on the performance of a specific model (e.g., accuracy, AUC). We evaluated three prominent wrapper strategies in this experiment: Recursive Feature Elimination (RFE), Sequential Forward Selection (SFS), and Sequential Backward Elimination (SBE).

These methods offer significant advantages as they consider feature interactions and are oriented towards the performance of a specific model, often yielding the best-performing feature subset for that particular model. However, they also present notable disadvantages, being computationally extremely expensive due to the need for repeated model training. Additionally, they carry a higher risk of overfitting to the training data, requiring rigorous cross-validation to ensure robust results.

5.3.1 Recursive Feature Elimination (RFE)

RFE starts with the full set of features and iteratively removes the least important feature (based on model coefficients or feature importances) until the desired number of features is reached. The elimination order reveals the feature ranking.

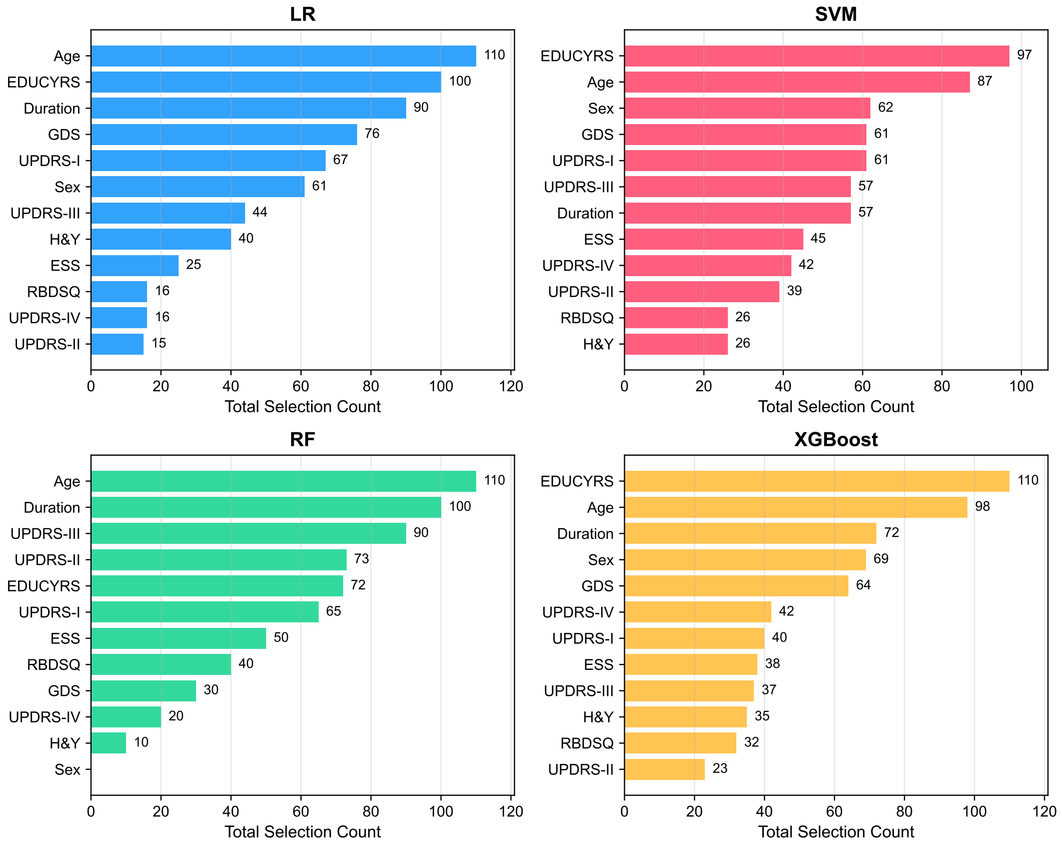

Figure S10. Feature importance ranking from Recursive Feature Elimination (RFE) across four machine learning models. The count represents how many times each feature was retained during the elimination process across 10-fold cross-validation with subject-level stratified sampling within the training dataset. Higher counts indicate greater feature importance, as these features were eliminated later in the process.

This experiment was conducted using 10-fold cross-validation with subject-level stratified sampling within the training dataset. For each fold of cross-validation, we recorded the number of times each feature was selected. Starting from the original 12 features, RFE was used to progressively eliminate features until only one feature remained. For each feature, the results from the 11 RFE iterations were aggregated, yielding the results presented in Figure S10.

The overall RFE results highlight Age, EDUCYRS, and Duration as the most critical features. For LR and XGBoost, all three features (Age, EDUCYRS, and Duration) consistently rank within the top three positions. For SVM, Age and EDUCYRS rank in the top two positions, while Duration ranks seventh. For RF, Age and Duration occupy the top two positions, with EDUCYRS ranking fifth. Despite some model-specific variations, Age, EDUCYRS, and Duration emerge as the most critical features overall, demonstrating high retention rates across cross-validation folds. While the importance of these top-ranked features remains stable across different models and validation folds, there is greater variability among the lower-ranked features, indicating their lesser predictive contribution or model-dependent utility.

5.3.2 Sequential Forward Selection (SFS)

SFS starts with an empty set of features and iteratively adds the feature that results in the highest performance improvement for the model. The selection order determines the feature ranking, with lower selection order values indicating earlier selection and higher feature importance.

This experiment was conducted using 10-fold cross-validation with subject-level stratified sampling within the training dataset. In the first iteration, all 12 features were evaluated, and the feature yielding the highest AUC-PR value was selected and retained. Subsequently, in each iteration, one additional feature from the remaining candidates that achieved the highest AUC-PR improvement was added to the selected set. This process continued until all features were ranked according to their selection order, as illustrated in Figure S11.

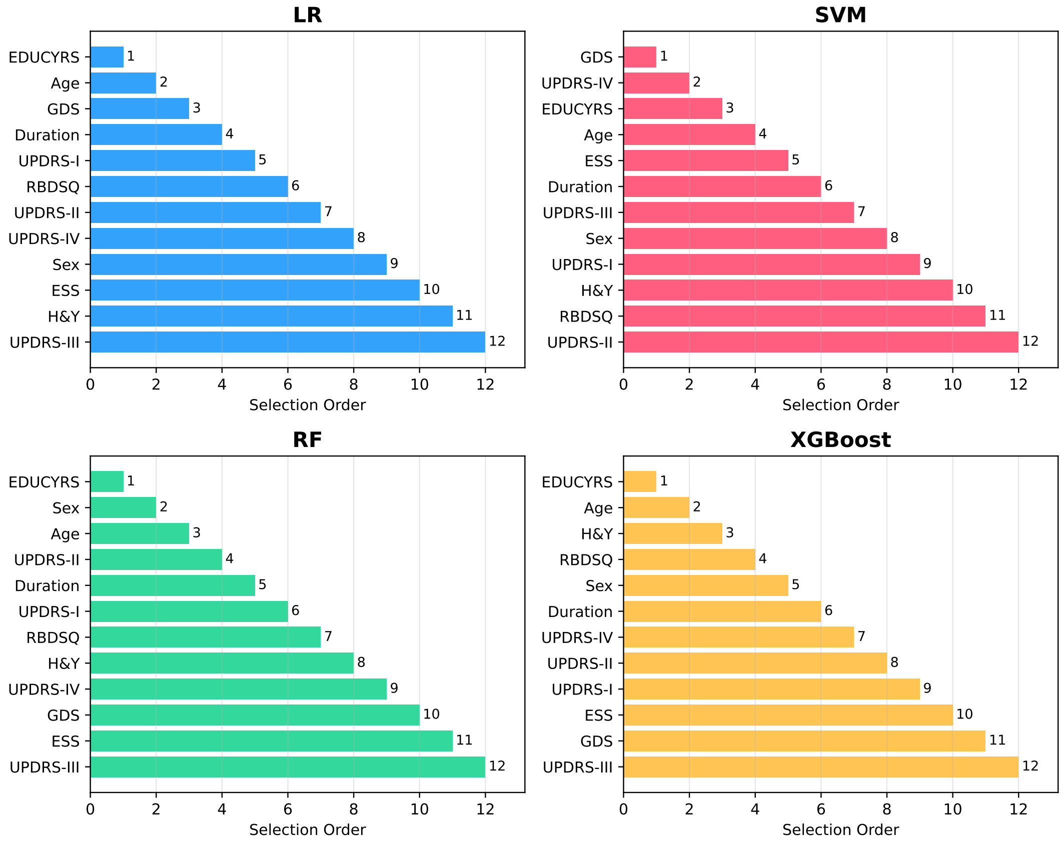

Figure S11. Feature selection order from Sequential Forward Selection (SFS) across four machine learning models, where lower values indicate earlier selection and higher feature importance. Results were obtained using 10-fold cross-validation with subject-level stratified sampling in the training set.

The SFS results demonstrate remarkable consistency in identifying the most predictive features. For three algorithms (LR, RF, and XGBoost), both EDUCYRS and Age consistently rank within the top three most important features (lowest selection order values). For SVM, these two features rank third and fourth respectively, showing only slight variation. Overall, EDUCYRS and Age emerge as the most critical features identified through SFS, which aligns excellently with the findings from our main experiment.

The performance curve in Figure S12 provides additional insights, showing that for most models, the AUC-PR score peaks or plateaus after selecting approximately 7-8 features. This demonstrates a clear point of diminishing returns, suggesting that the remaining features contribute minimal additional predictive value beyond this core set of highly informative variables.

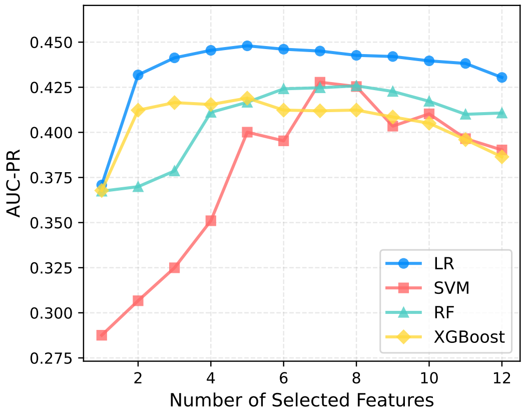

Figure S12. AUC-PR performance as features are sequentially added using SFS. Performance tends to plateau after a certain number of features are included, demonstrating diminishing returns beyond the core feature set.

5.3.3 Sequential Backward Elimination (SBE)

SBE operates as the inverse of SFS, beginning with the complete feature set and iteratively eliminating features whose removal results in the smallest performance degradation. This experiment employed 10-fold cross-validation with subject-level stratified sampling within the training dataset.

The elimination process follows a systematic approach: starting with all 12 features, each feature is evaluated for removal, and the feature whose elimination yields the smallest decrease in AUC-PR performance (or potentially an improvement) is removed from the active set. This iterative process continues, with one feature eliminated per iteration based on the criterion of minimal performance impact, until only a single feature remains. The order of elimination provides the feature ranking, with features eliminated later considered more important, as demonstrated in Figure S13.

The SBE results demonstrate remarkable consistency with both RFE and SFS findings. For all four models (LR, RF, SVM, and XGBoost), EDUCYRS and Age consistently rank among the last features to be eliminated, confirming their critical importance and aligning perfectly with the main experiment results. Duration also shows high retention across models, further validating the core feature set identified in our primary analysis.

The performance curve in Figure S14 reveals that model performance remains stable or declines slowly during the initial elimination phases until the most critical features are removed, at which point performance drops sharply. This pattern validates the indispensable nature of the core feature set (Age, EDUCYRS, and Duration) and demonstrates clear diminishing returns when these essential predictors are eliminated from the model.

5.4 Embedded Methods

Embedded methods integrate the feature selection process directly into the model training algorithm. The model learns which features are most important as part of its construction. Regularization methods like LASSO are a prime example. These methods offer several advantages: they are computationally more efficient than wrapper methods, consider feature interactions, and are often less prone to overfitting due to built-in regularization. However, they also have limitations, as the feature selection is specific to the model used.

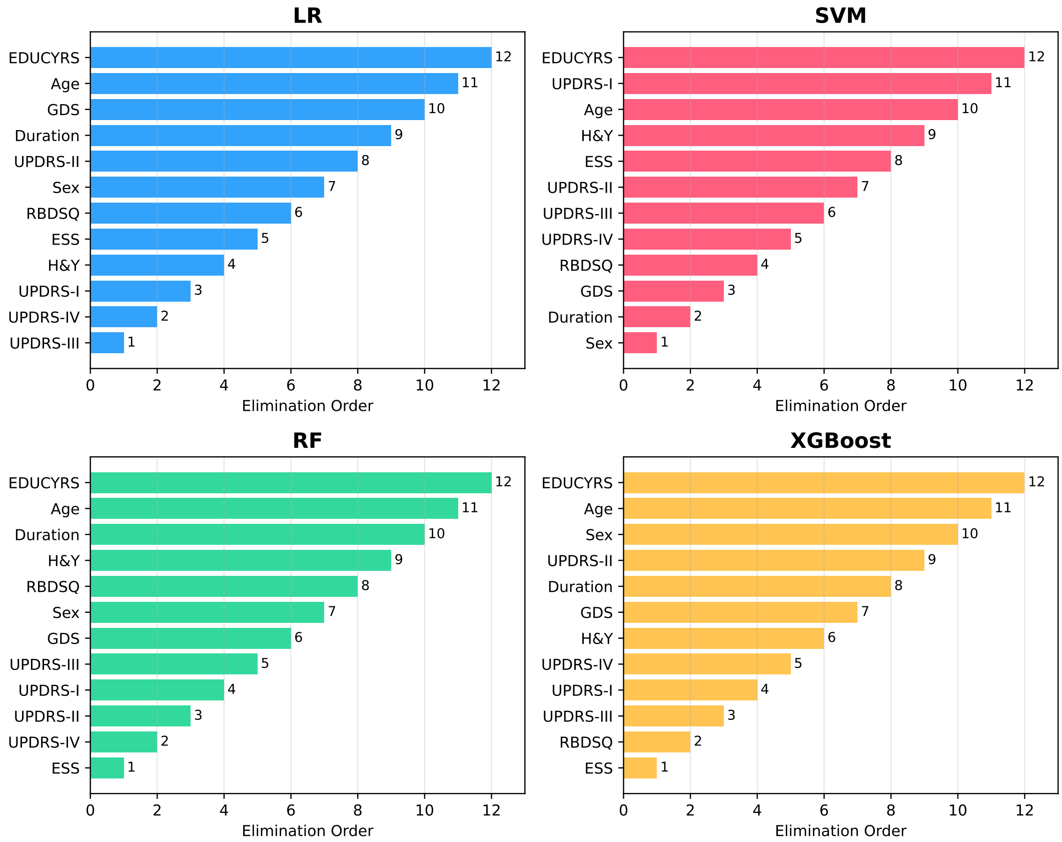

Figure S13. Feature elimination order from Sequential Backward Elimination (SBE) across four machine learning models. Higher elimination order values indicate later elimination and greater feature importance. Results were obtained using 10-fold cross-validation with subject-level stratified sampling in the training set, where features eliminated last are considered most critical for model performance.

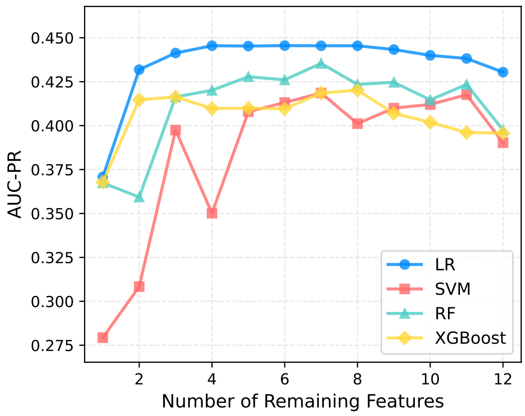

Figure S14. AUC-PR performance trajectory as features are sequentially eliminated using SBE. The curve demonstrates that model performance remains stable during initial elimination phases until critical features are removed, at which point performance drops sharply, highlighting the indispensable nature of core predictive features.

It is important to note that the feature ranking methods employed in Supplementary Experiment II are essentially embedded methods. However, in Supplementary Experiment II, hyperparameter optimization was performed for each model to achieve optimal performance. In contrast, the wrapper methods in

Supplementary Experiment IV, due to their computational complexity and methodological demands, did not include fine-tuned hyperparameter optimization for each model. To enable direct comparison with the wrapper method experiments in Supplementary Experiment IV, this experiment builds upon Supplementary Experiment II by using default parameters for each model without hyperparameter tuning, and then ranking features based on their intrinsic importance scores.

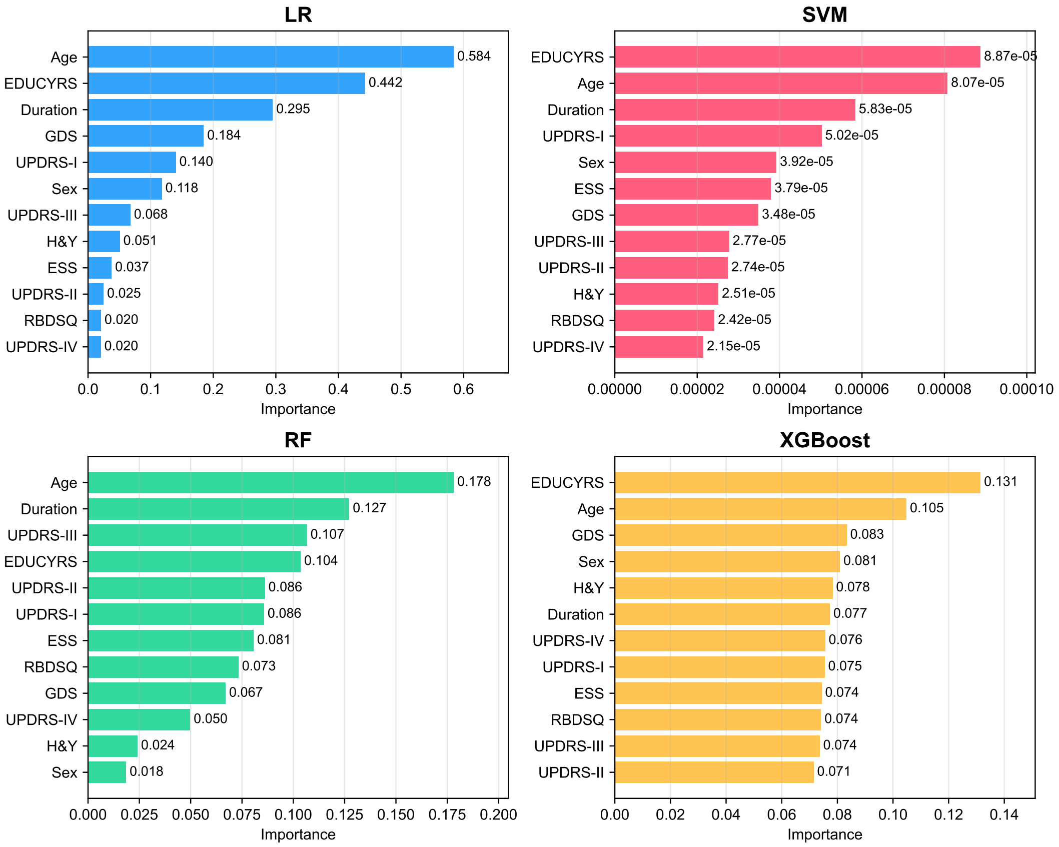

Figure S15. Feature importance ranking from embedded methods across four machine learning models using default parameters. The visualization displays feature importance scores derived from each model's intrinsic importance calculations, where higher values indicate greater feature importance. Results were obtained using 10-fold cross-validation with subject-level stratified sampling in the training dataset.

The results from the four embedded models using default parameters are shown in Figure S15. Despite the differences in how each algorithm calculates importance (e.g., coefficients for LR, Gini impurity for RF), there is a strong consensus that aligns remarkably well with the main experiment findings.

For LR and SVM, Age, EDUCYRS, and Duration consistently occupy the top three positions. For RF, Age and Duration rank in the top two positions, with EDUCYRS ranking fourth. For XGBoost, EDUCYRS and Age secure the top two positions. These results demonstrate high consistency with the findings from the main experiment, further validating the robustness of these core predictive features.

5.5 Comprehensive Comparison Across Methods

To provide a holistic view of feature importance across all methodologies, we present a comprehensive comparison that integrates the results from filters methods, three wrapper methods (RFE, SFS, SBE) and the embedded methods.

The comprehensive comparison across all methods (Figures S16) demonstrates remarkable consistency in feature importance rankings. This cross-methodological validation strengthens the evidence for the critical role of Age, EDUCYRS, and Duration as the most predictive features for PD-MCI classification. The convergence of results across fundamentally different algorithmic approaches provides robust statistical evidence for the reliability and generalizability of these findings.

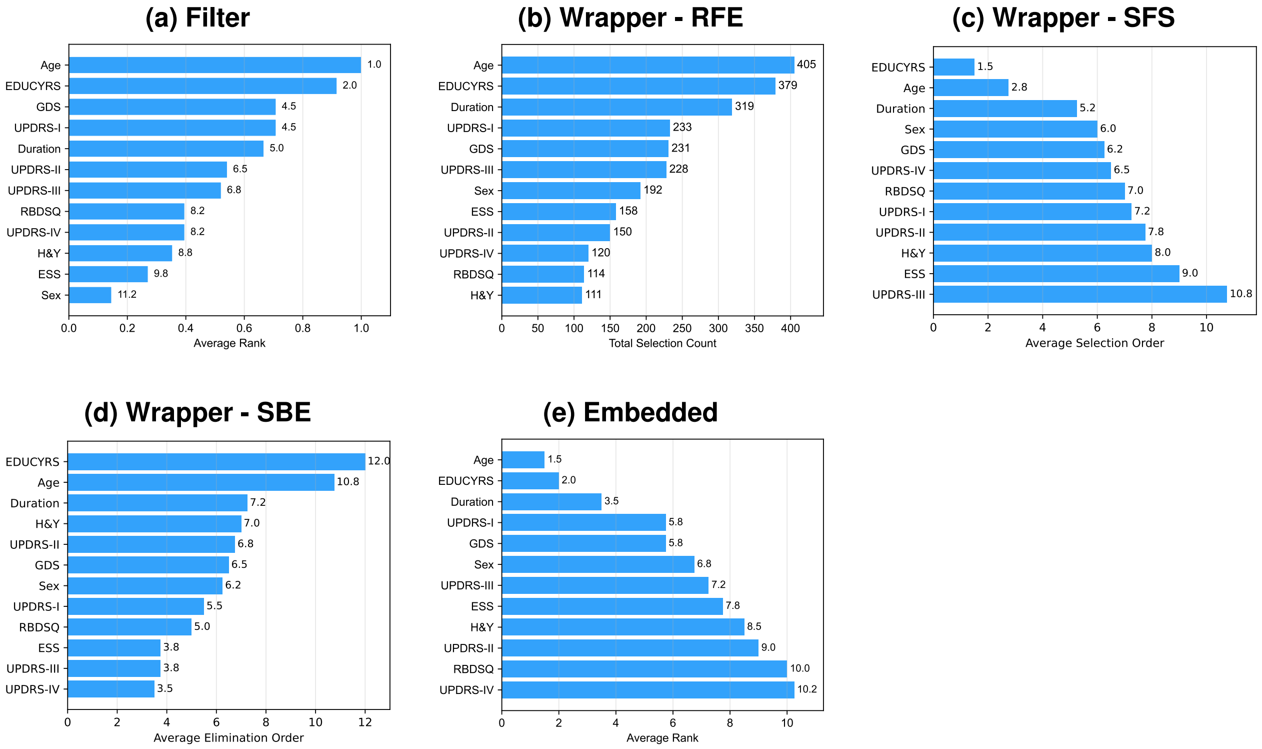

Figure S16. Comprehensive comparison of feature importance across all filter, wrapper and embedded methods: (a) Filter methods showing average normalized feature importance scores, (b) Recursive Feature Elimination (RFE) showing total selection count, (c) Sequential Forward Selection (SFS) demonstrating average selection order, (d) Sequential Backward Elimination (SBE) showing average elimination order, and (e) Average feature ranking from embedded methods. In all visualizations, features displayed higher in the plots indicate greater importance.

To provide a comprehensive visualization of the cross-methodological consensus, Figure S17 presents a comparative ranking heatmap that consolidates the results from all 20 feature selection approaches across the five methodological categories. This visualization clearly demonstrates the remarkable consistency in feature importance patterns across fundamentally different algorithmic approaches.

The complete source code for this experiment is available at: `https://github.com/yuzhounh/PD-MCI-Classification/tree/main/supplementary_experiment_4`.

5.6 Conclusion

This comprehensive analysis reveals a powerful and consistent narrative. Across three fundamentally different families of feature selection methods—model-agnostic filters, performance-driven wrappers, and model-integrated embedded techniques—a core set of features consistently emerges as the most predictive. Age, EDUCYRS, and Duration are unequivocally the most dominant predictors for PD-MCI classification. This cross-methodological consensus provides the highest level of confidence in their statistical and clinical

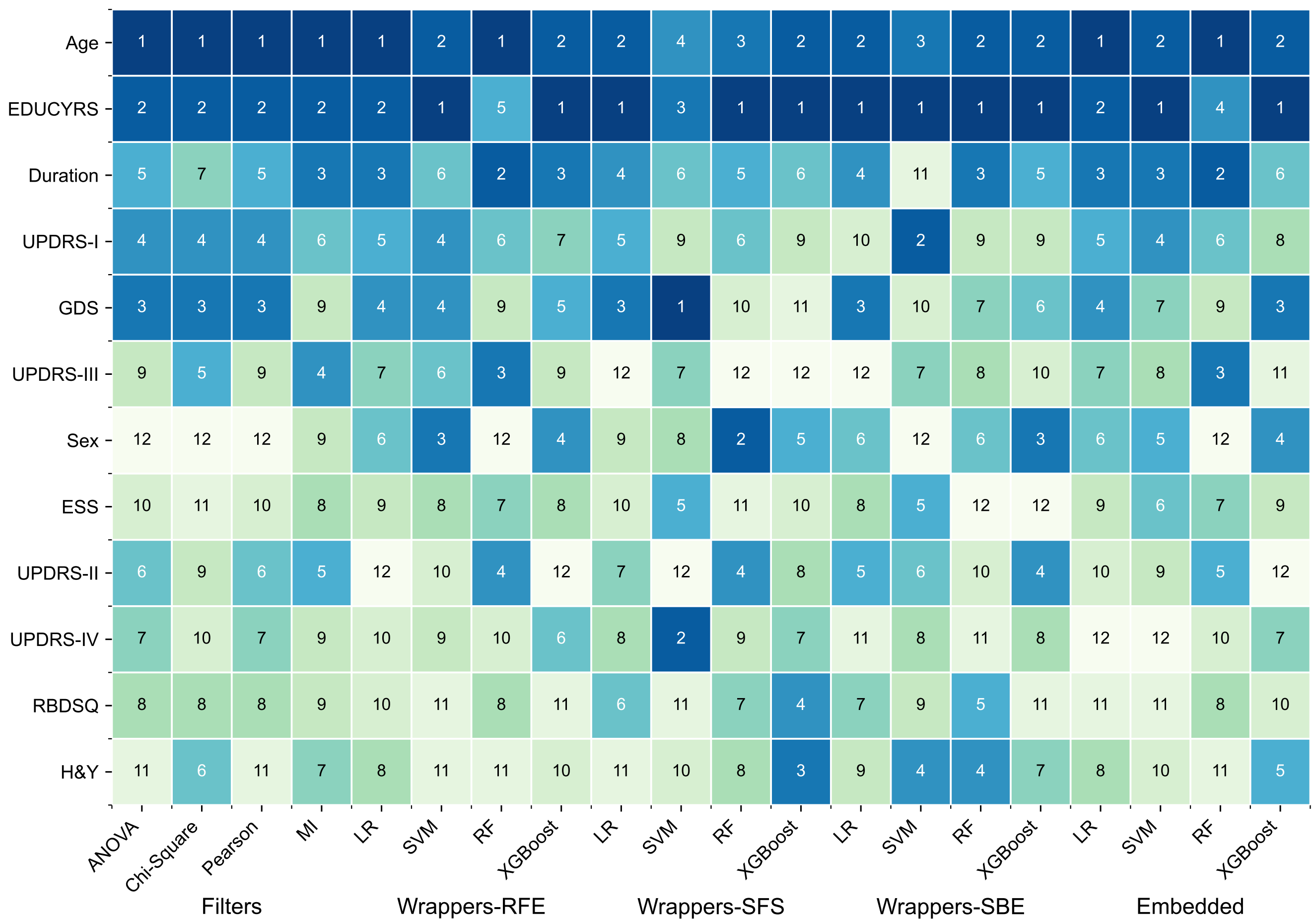

Figure S17. Comparative feature ranking heatmap across 20 feature selection methods from five categories (Filters, Wrappers-RFE, Wrappers-SFS, Wrappers-SBE, and Embedded methods). Numbers indicate ranking (1 = most important, 12 = least important), with color intensity reflecting importance levels. Age, EDUCYRS, and Duration consistently emerge as top-ranked features across all methodological approaches.

relevance, suggesting that a parsimonious model built upon these features is likely to be both robust and generalizable.
